# ESCO1 stabilizes NUFIP2 through acetylation–ubiquitination crosstalk to promote pancreatic cancer progression

**DOI:** 10.3389/fimmu.2026.1929713

**Published:** 2026-10-02

**Authors:** Letian Chen, Zihan Yan, Yuting Rao, Xiaowei Fu, Keyi Fang, Zijian Zhou, Xiaogang Peng, Yeqing Zou

**Affiliations:** 1 Jiangxi Key Laboratory of Molecular Medicine, The Second Affiliated Hospital, Jiangxi Medical College, Nanchang University, Nanchang, Jiangxi, China; 2 Department of Hepatobiliary and Pancreatic Surgery, The Second Affiliated Hospital, Jiangxi Medical College, Nanchang University, Nanchang, Jiangxi, China

**Keywords:** ESCO1, malignant tumor progression, mitochondrial apoptosis, NUFIP2, pancreatic cancer

## Abstract

**Background:**

Pancreatic cancer is a highly aggressive malignancy with a poor prognosis, and its molecular pathogenesis remains incompletely understood, with effective therapeutic options still limited. Establishment of sister chromatid cohesion N-acetyltransferase 1 (ESCO1) has been implicated in the development and progression of several malignancies; however, its biological role and underlying mechanisms in pancreatic cancer remain largely unknown. This study aimed to elucidate the clinical significance and molecular mechanisms of the ESCO1/NUFIP2 axis in pancreatic cancer.

**Methods:**

TCGA, CPTAC, and other publicly available datasets were used to evaluate the expression patterns, diagnostic value, and prognostic significance of ESCO1 and NUFIP2 in pancreatic cancer. *In vitro* and *in vivo* experiments were subsequently performed to characterize the biological functions of the ESCO1/NUFIP2 axis and elucidate its underlying molecular mechanisms.

**Results:**

ESCO1 and NUFIP2 were significantly upregulated in pancreatic cancer and were associated with poor prognosis. Functional assays demonstrated that the ESCO1/NUFIP2 axis promoted pancreatic cancer cell proliferation and migration, enhanced tumor growth *in vivo*, and suppressed apoptosis. Mechanistically, ESCO1 promoted NUFIP2 acetylation and limited its K48-linked ubiquitination and subsequent degradation, thereby maintaining NUFIP2 protein stability. Stabilized NUFIP2 preserved mitochondrial function and suppressed mitochondrial apoptosis, whereas NUFIP2 overexpression or trichostatin A (TSA) treatment partially reversed the effects of ESCO1 depletion. Moreover, the ESCO1/NUFIP2 axis was associated with immune cell infiltration, and depletion of either ESCO1 or NUFIP2 enhanced dendritic cell (DC)-mediated cytotoxic T lymphocyte (CTL) antitumor responses.

**Conclusion:**

ESCO1 stabilizes NUFIP2 through acetylation-dependent regulation, thereby preserving mitochondrial function, suppressing mitochondrial apoptosis, and promoting pancreatic cancer progression. The ESCO1/NUFIP2 axis may also contribute to the regulation of antitumor immunity, highlighting its potential as a biomarker and therapeutic target in pancreatic cancer.

## 1 Introduction

Pancreatic cancer is one of the most aggressive and lethal malignancies of the digestive system, characterized by rapid progression, insidious onset, and a lack of specific clinical manifestations. Most patients are diagnosed with locally advanced or metastatic disease, posing substantial challenges to effective clinical management (1). Despite advances in surgical resection, chemotherapy, targeted therapy, and immunotherapy, the overall prognosis of patients with pancreatic cancer remains poor. Moreover, pancreatic cancer is characterized by a profoundly immunosuppressive tumor microenvironment, in which impaired effector immune cell activity and complex tumor–stromal interactions further constrain effective antitumor immune responses (2). Therefore, elucidating the molecular mechanisms underlying pancreatic cancer progression and their potential links to the tumor immune microenvironment is critical for identifying novel biomarkers and therapeutic targets.

Protein acetylation is an important post-translational modification that regulates diverse biological processes, including cell proliferation, apoptosis, DNA damage repair, and metabolic reprogramming, by modulating protein stability, subcellular localization, molecular interactions, and functional activity (3). Aberrant protein acetylation has been implicated in the initiation and progression of various malignancies and can profoundly influence tumor cell behavior and therapeutic responses (4–6). Establishment of sister chromatid cohesion N-acetyltransferase 1 (ESCO1) is an acetyltransferase whose canonical functions involve sister chromatid cohesion, DNA replication, and the maintenance of genomic stability (7, 8). However, the biological functions of ESCO1 may extend beyond its canonical role in chromosome regulation, as acetylation of non-histone substrates can also modulate protein stability and cell fate. Nevertheless, the biological significance and potential oncogenic mechanisms of ESCO1 in pancreatic cancer remain poorly understood, particularly whether ESCO1 contributes to tumor progression by regulating the post-translational modification and stability of non-histone substrates.

Analysis of the potential downstream regulatory network of ESCO1 identified NUFIP2 as a candidate effector. NUFIP2 is an RNA-binding protein that recognizes G-rich RNA sequences and participates in RNA granule transport, RNA localization, and RNA homeostasis (9). Accumulating evidence indicates that RNA-binding proteins not only regulate RNA metabolism but also contribute to tumor cell proliferation, metastasis, and cell death by modulating gene expression, proteostasis, and cellular stress responses (10, 11). However, the role of NUFIP2 in cancer remains largely unexplored, and its biological function and upstream regulatory mechanisms in pancreatic cancer are unknown. Given the potential molecular association between ESCO1 and NUFIP2, we sought to determine whether NUFIP2 functions as a downstream effector of ESCO1 and whether ESCO1 regulates NUFIP2 protein stability through its acetyltransferase activity, thereby contributing to pancreatic cancer progression.

In this study, we integrated public database analyses, clinical tissue validation, and *in vitro* and *in vivo* experiments to systematically characterize the expression patterns, clinical significance, and biological functions of ESCO1 and NUFIP2 in pancreatic cancer and to elucidate the underlying molecular mechanisms. We found that ESCO1 promotes NUFIP2 acetylation and limits its K48-linked ubiquitination and subsequent degradation, thereby maintaining NUFIP2 protein stability. Stabilized NUFIP2, in turn, preserves mitochondrial function, suppresses mitochondrial apoptosis, and promotes pancreatic cancer progression. Furthermore, immune-related analyses and DC–CTL functional assays suggest that the ESCO1/NUFIP2 axis may also contribute to the regulation of antitumor immune responses. Collectively, these findings establish the ESCO1/NUFIP2 axis as a previously unrecognized oncogenic regulatory pathway in pancreatic cancer and suggest a potential link between tumor cell-intrinsic survival and antitumor immunity, providing new mechanistic insights into pancreatic cancer progression.

## 2 Materials and methods

### 2.1 Bioinformatics analysis

#### 2.1.1 Expression analysis of ESCO1 across pan-cancer, normal tissues, and single-cell types

The expression levels of ESCO1 across 33 tumor types and their corresponding normal tissues were analyzed using data from The Cancer Genome Atlas (TCGA). Because normal control samples are absent or limited for certain tumor types in the TCGA dataset, the GEPIA2 platform, which integrates TCGA and Genotype-Tissue Expression (GTEx) data, was further used for complementary validation to increase the number of normal control samples and improve the reliability of the analysis. In addition, the Human Protein Atlas (HPA) database was used to investigate the mRNA expression patterns of ESCO1 across different normal tissues and cell types.

#### 2.1.2 Immune associations and prognostic significance of ESCO1 across pan-cancer

To evaluate the immune-related characteristics of ESCO1 across different cancer types, pan-cancer transcriptomic data were integrated with immune cell infiltration data, and the correlations between ESCO1 expression and the infiltration levels of different immune cell populations were analyzed. The results were visualized using the ggplot2 package (v3.4.4) in R.

To further assess the prognostic significance of ESCO1 across different tumor types, transcriptomic expression data and corresponding clinical follow-up information were obtained from the TCGA database. Samples lacking survival time or survival status information were excluded. Kaplan–Meier survival analysis and univariate Cox proportional hazards regression analysis were performed using the survival and survminer packages in R (v4.3.0) to evaluate the association between ESCO1 expression and overall survival (OS).

#### 2.1.3 Expression, diagnostic value, and prognostic significance of ESCO1 and NUFIP2 in pancreatic cancer

The mRNA expression levels of ESCO1 and NUFIP2 in pancreatic cancer tissues and normal pancreatic tissues were analyzed using TCGA data, and their protein expression patterns were further evaluated using data from the Clinical Proteomic Tumor Analysis Consortium (CPTAC). Receiver operating characteristic (ROC) curve analysis was performed to assess the diagnostic performance of ESCO1 and NUFIP2 for pancreatic cancer. Sample type (tumor or normal) was used as the classification variable, whereas the expression level of ESCO1 or NUFIP2 was used as the predictor variable. The area under the curve (AUC) was calculated to evaluate diagnostic performance.

Transcriptomic expression data and corresponding clinical follow-up information for patients with pancreatic cancer were subsequently obtained from the TCGA database. Samples with missing survival time or survival status were excluded. Kaplan–Meier survival analysis and univariate Cox proportional hazards regression analysis were performed using the survival and survminer packages to evaluate the associations of ESCO1 and NUFIP2 expression with OS. Univariate and multivariate Cox proportional hazards regression models were further constructed by incorporating clinicopathological variables, including age, sex, pathological stage, T stage, and N stage, to assess whether ESCO1 and NUFIP2 were independent prognostic factors in pancreatic cancer. The results were visualized using forest plots.

#### 2.1.4 Screening of ESCO1-related genes and molecular docking analysis of ESCO1 and NUFIP2 in pancreatic cancer

Pearson correlation analysis was performed using pancreatic cancer transcriptomic data to systematically evaluate the associations between ESCO1 expression and the expression levels of other genes. Candidate genes significantly correlated with ESCO1 were screened according to the correlation coefficient and statistical significance. On this basis, candidate genes were further evaluated by integrating expression correlation, biological function, and potential research relevance, and NUFIP2 was selected as a potential downstream molecule of ESCO1. The expression correlation between ESCO1 and NUFIP2 was subsequently analyzed in greater detail.

To preliminarily predict the potential protein–protein interaction between ESCO1 and NUFIP2, molecular docking analysis was performed using the HDOCK online platform. Candidate complex conformations were selected according to docking scores and predicted binding modes, and representative docking models and potential interaction interfaces were visualized using PyMOL.

#### 2.1.5 Associations of ESCO1 and NUFIP2 with the tumor microenvironment in pancreatic cancer

Pancreatic cancer samples were divided into ESCO1-high and ESCO1-low expression groups according to the median ESCO1 expression level. The ESTIMATE algorithm was used to characterize the tumor microenvironment of each sample and to calculate the Immune Score, Stromal Score, and ESTIMATE Score. Differences in these scores between the ESCO1-high and ESCO1-low groups were compared using the Wilcoxon rank-sum test and visualized using violin plots.

The correlations between the expression levels of ESCO1 or NUFIP2 and the infiltration levels of different immune cell populations were further analyzed. The strength of the associations was evaluated according to the correlation coefficients and corresponding statistical significance, and the results were visualized using lollipop plots in R.

#### 2.1.6 Expression of ESCO1 and NUFIP2 and cell–cell communication analysis in pancreatic cancer single-cell RNA sequencing data

The pancreatic cancer single-cell RNA sequencing dataset GSE205013 was downloaded from the Gene Expression Omnibus (GEO) database. Data quality control, normalization, dimensionality reduction, clustering, and cell-type annotation were performed using the Seurat package (v4.0) in R. The quality-control criteria were as follows: mitochondrial gene percentage <20%, number of detected genes per cell between 200 and 6,000, and UMI count >500. After quality control, the data were normalized using the LogNormalize method, followed by principal component analysis (PCA). UMAP dimensionality reduction and cell clustering were performed based on the first 30 principal components. Major cell populations were annotated according to established canonical marker genes.

Cell–cell communication analysis was performed using CellPhoneDB to predict potential ligand–receptor interactions among different cell populations. FeaturePlot and violin plots were used to visualize the expression distributions of ESCO1 and NUFIP2 across different cell types.

### 2.2 Patients and tissue specimens

A total of 33 patients with pancreatic cancer who underwent radical surgical resection at the Department of General Surgery, the Second Affiliated Hospital of Nanchang University, were enrolled in this study. Surgically resected pancreatic cancer tissues and corresponding clinicopathological data were collected. None of the patients had received radiotherapy, chemotherapy, or other antitumor treatments before surgery. This study was reviewed and approved by the Ethics Committee of the Second Affiliated Hospital of Nanchang University and was conducted in accordance with the principles of the Declaration of Helsinki. All tissue specimens were obtained during routine clinical care without additional interventions or risks to the patients; therefore, the requirement for written informed consent was waived with approval from the Ethics Committee.

### 2.3 Cell culture, lentiviral infection, and plasmid transfection

The human pancreatic ductal epithelial cell line HPDE6-C7 and the human pancreatic cancer cell lines PANC-1, SW1990, ASPC-1, and MIAPaCa-2 were purchased from Procell Life Science & Technology Co., Ltd. (Wuhan, China). All cell lines were authenticated by short tandem repeat (STR) profiling and confirmed to be free of mycoplasma contamination. Cells were cultured in the corresponding complete culture medium supplemented with 10% fetal bovine serum and maintained in a humidified incubator at 37 °C with 5% CO_2_.

shRNA vectors targeting ESCO1 and NUFIP2, as well as the NUFIP2 overexpression plasmid, were constructed by Sangon Biotech (Shanghai) Co., Ltd. Three ESCO1-specific shRNAs were designed with the following target sequences: shESCO1-1, 5′-CGGAGATCACAAAGGCTACAA-3′; shESCO1-2, 5′-GCCTAAGTGTAATTCCCATTT-3′; and shESCO1-3, 5′-CCAGCTAAATAGGAAGAATTA-3′. Three NUFIP2-specific shRNAs were designed with the following target sequences: shNUFIP2-1, 5′-GAACAAATTAAGACTAGCCTT-3′; shNUFIP2-2, 5′-CGGTTACATATTGACTTTAA-3′; and shNUFIP2-3, 5′-GAAACTTTCAAGCCTGACTAT-3′.

Lentiviral particles carrying the shRNA constructs were produced in 293T cells. When 293T cells reached 80–90% confluence, plasmid transfection was performed using PEI transfection reagent (G1802, Servicebio) according to the manufacturer’s instructions. pMDL (1.16 μg), pVSVG (0.62 μg), pREV (0.45 μg), and the corresponding shRNA vector were mixed thoroughly with PEI and added to the cells, with a final culture volume of 2 mL per well. Cells were then incubated at 37 °C in 5% CO_2_ (12). At 48 h after transfection, the culture supernatants were collected and centrifuged at 300–500 × g for 5 min to remove cellular debris, followed by filtration through a 0.45-μm membrane. The resulting filtrates containing lentiviral particles were used to infect PANC-1 and SW1990 cells. At 48–72 h after infection, cells were harvested, and ESCO1 or NUFIP2 protein expression was examined by Western blotting to confirm knockdown efficiency.

The NUFIP2 overexpression plasmid was generated by amplifying the full-length coding sequence (CDS) of NUFIP2 and cloning it into the pcDNA3.1 vector. The cloning primers were as follows: forward, 5′-CTAGCTAGCATGGAGGAGAAGCCCGGCCA-3′; reverse, 5′-CGGGGTACCTCATTGATCTGGACTATCCA-3′. The sequence-verified pcDNA3.1-NUFIP2 plasmid was directly transfected into PANC-1 and SW1990 cells using a transfection reagent, with the corresponding empty vector used as the negative control. NUFIP2 protein expression was subsequently examined by Western blotting to confirm overexpression efficiency.

### 2.4 Quantitative real-time PCR

Cells were harvested when they reached 70–90% confluence. Total RNA was extracted using an RNA Rapid Purification Kit (Beyotime Biotechnology, Shanghai, China). Complementary DNA (cDNA) was synthesized using the PrimeScript™ RT Reagent Kit (Takara Bio Inc., Shiga, Japan). Quantitative real-time PCR (qRT-PCR) was performed using TB Green^®^ Premix Ex Taq™ II (Takara Bio Inc., Shiga, Japan). GAPDH was used as the internal reference gene, and relative gene expression levels were calculated using the 2^−ΔΔCt^ method (13).

All primers were synthesized by Sangon Biotech (Shanghai) Co., Ltd. The primer sequences were as follows: ESCO1 forward, 5′-GCCAGAATTGGAAACACGCA-3′ and reverse, 5′-TCCCCTCACAGTCACCGTAT-3′; NUFIP2 forward, 5′-ACCACCATCATCACCAGCAG-3′ and reverse, 5′-GGAGGGTGTGTTTCTGCTCA-3′; and GAPDH forward, 5′-GGAGCGAGATCCCTCCAAAAT-3′ and reverse, 5′-GGCTGTTGTCATACTTCTCATGG-3′.

### 2.5 Western blotting

Cells were washed twice with ice-cold phosphate-buffered saline (PBS) and collected. Total protein was extracted using RIPA lysis buffer (Solarbio Science & Technology Co., Ltd., Beijing, China), and protein concentrations were determined using a BCA Protein Assay Kit (Solarbio Science & Technology Co., Ltd., Beijing, China). Equal amounts of protein (20 μg) were mixed with loading buffer, denatured at 100 °C for 10 min, separated by 10% SDS-PAGE, and transferred onto nitrocellulose (NC) membranes.

The membranes were blocked with 5% non-fat milk for 2 h at room temperature and then incubated overnight at 4 °C with primary antibodies against ESCO1 (1:1000, Cat. No. ab211475, Abcam, Cambridge, UK), NUFIP2 (1:1000, Cat. No. 10893, Cell Signaling Technology, Inc., Danvers, MA, USA), and GAPDH (1:40000, Cat. No. 10494-1-AP, Proteintech Group, Inc., Wuhan, China). After three washes with TBST (Servicebio, Wuhan, China), the membranes were incubated with HRP-conjugated secondary antibodies (1:7000, Cat. No. SA00001-2, Proteintech Group, Inc., Wuhan, China) for 1 h at room temperature. Protein bands were visualized using enhanced chemiluminescence (ECL) reagents (UElandy, Suzhou, China) and quantified using Image Lab software (Bio-Rad, USA). All experiments were independently repeated at least three times (14).

### 2.6 Co-immunoprecipitation

Co-immunoprecipitation (Co-IP) assays were performed to examine the interaction between ESCO1 and NUFIP2 and to assess the acetylation and ubiquitination levels of NUFIP2. Cells from the indicated treatment groups were harvested and lysed thoroughly in IP lysis buffer. After centrifugation, the supernatants were collected. Equal amounts of cell lysates were incubated overnight at 4 °C with the indicated specific antibodies or an isotype-matched IgG control, followed by incubation with Protein A/G magnetic beads (Beyotime Biotechnology, Shanghai, China) (15). The beads were extensively washed to remove nonspecifically bound proteins, and the immunoprecipitated complexes were then eluted and analyzed by Western blotting to detect the target proteins and corresponding post-translational modifications.

### 2.7 Cell proliferation assays

Colony formation and EdU incorporation assays were performed to evaluate the proliferative capacity of pancreatic cancer cells. For the colony formation assay, treated cells were seeded into 6-well plates at a density of 500 cells per well, and the culture medium was replaced every 3 days. After 14 days of culture, the colonies were fixed with 4% paraformaldehyde and stained with crystal violet. Images were captured, and colony numbers were quantified using ImageJ software.

For the EdU assay, the YF^®^ 488 Click-iT EdU staining kit (UElandy, Suzhou, China) was used. After incubation with EdU, the staining procedure was performed according to the manufacturer’s instructions, and nuclei were counterstained with Hoechst 33342. Images were acquired using an Olympus fluorescence microscope, and the proportion of EdU-positive cells was calculated using ImageJ software.

### 2.8 Cell migration and invasion assays

Wound-healing and Transwell assays were performed to evaluate the migratory and invasive abilities of pancreatic cancer cells. For the wound-healing assay, treated cells were seeded into 6-well plates at a density of 4 × 10^5^ cells per well. When cell confluence reached approximately 90%, a uniform scratch was generated across the cell monolayer using a sterile 200-μL pipette tip. Detached cells and debris were removed by gently washing the cells twice with PBS, after which serum-free medium was added. Images of the same wound areas were captured at 0, 24, and 48 h, and cell migration was evaluated based on changes in the wound area.

For the Transwell migration assay, 2 × 10^4^ treated cells were seeded into the upper chambers of Transwell inserts with 8-μm pores in serum-free medium, whereas complete medium containing 10% fetal bovine serum was added to the lower chambers as a chemoattractant. For the invasion assay, the upper chambers were precoated with Matrigel before cell seeding. After 24 h of incubation, cells remaining on the upper surface of the membrane were gently removed with a cotton swab. Cells that had migrated or invaded to the lower surface of the membrane were fixed with 4% paraformaldehyde and stained with crystal violet. Images were acquired under a light microscope, and the number of migrated or invaded cells was quantified using ImageJ software.

### 2.9 Flow cytometric analysis of apoptosis

Cell apoptosis was assessed using Annexin V-FITC/PI double staining(UElandy, Suzhou, China). Cells from each group were harvested, washed with PBS, and resuspended in binding buffer at a concentration of 1 × 10^6^ cells/mL. A 100-μL aliquot of the cell suspension was incubated with 5 μL Annexin V-FITC and 10 μL propidium iodide (PI) for 15 min at room temperature in the dark (16). Samples were subsequently analyzed using a FACScan flow cytometer (BD Biosciences), and the proportions of early and late apoptotic cells were quantified using FlowJo software (version 10, FlowJo LLC).

### 2.10 Subcutaneous xenograft tumor model

Stably transfected pancreatic cancer cells were harvested, counted, and resuspended in sterile PBS before subcutaneous injection into BALB/c nude mice. Tumor length and width were measured at regular intervals after inoculation, and tumor growth was recorded. At the predetermined experimental endpoint, the mice were euthanized, and the tumors were completely excised, weighed, and photographed. Portions of the tumor tissues were fixed for subsequent immunohistochemical analysis. All animal experiments were reviewed and approved by the relevant institutional ethics committee and were conducted in accordance with applicable ethical guidelines for animal research.

### 2.11 Immunohistochemistry

Paraffin-embedded tumor tissue sections were sequentially deparaffinized, rehydrated, and subjected to antigen retrieval. Endogenous peroxidase activity was blocked with 3% hydrogen peroxide, and nonspecific binding sites were blocked with BSA. The sections were then incubated overnight at 4 °C with primary antibodies against ESCO1(1:500), NUFIP2(1:500), BCL-2 (1:500, Cat. No. 12789-1-AP, Proteintech Group, Inc., Wuhan, China), or Ki-67 (1:500, Cat. No. 27309-1-AP, Proteintech Group, Inc., Wuhan, China). The following day, the sections were incubated with the appropriate secondary antibodies, developed with DAB, counterstained with hematoxylin, dehydrated, cleared, and mounted. Images were acquired under a light microscope (17).

### 2.12 Confocal immunofluorescence and protein colocalization analysis

Treated PANC-1 and SW1990 cells were seeded into confocal culture dishes. After cell attachment, the cells were fixed with 4% paraformaldehyde and permeabilized with 0.3% Triton X-100. Following blocking with 5% BSA, cells were incubated overnight at 4 °C with primary antibodies against ESCO1 and NUFIP2. The next day, cells were incubated with species-appropriate fluorescently labeled secondary antibodies in the dark, followed by nuclear counterstaining with DAPI (18). Images were acquired using a laser scanning confocal microscope to examine the subcellular localization of ESCO1 and NUFIP2 and to evaluate their spatial colocalization. The NUFIP2 antibody used for immunofluorescence was purchased from Proteintech Group, Inc. (Cat. No. 67195-1-Ig, Wuhan, China).

### 2.13 Protein stability assay

A cycloheximide (CHX; Cat. No. HY-12320, MedChemExpress) chase assay was performed to evaluate NUFIP2 protein stability. Pancreatic cancer cells from the indicated treatment groups were cultured to approximately 80% confluence and then treated with 100 μg/mL CHX to inhibit *de novo* protein synthesis. Total protein was collected at 0, 4, 8, 12, 16, and 24 h after CHX treatment (19). NUFIP2 protein levels at each time point were examined by Western blotting, with GAPDH used as the loading control. Relative NUFIP2 protein levels were normalized to GAPDH and used to evaluate the degradation rate and protein stability of NUFIP2.

### 2.14 Cytochrome c redistribution and detection of mitochondrial apoptosis-related proteins

Confocal immunofluorescence microscopy was used to examine the subcellular distribution of cytochrome c (Cyt c). Pancreatic cancer cells from the indicated treatment groups were seeded into confocal culture dishes. After attachment, the cells were fixed with 4% paraformaldehyde, permeabilized with 0.3% Triton X-100, and blocked with 5% BSA. Cells were then incubated overnight at 4 °C with an anti-Cytochrome c primary antibody (1:200, Cat. No. 10993-1-AP, Proteintech Group, Inc., Wuhan, China). The following day, cells were incubated with the corresponding fluorescently labeled secondary antibody in the dark and counterstained with DAPI. Images were acquired using a laser scanning confocal microscope. Changes in Cyt c distribution from a punctate pattern to a more diffuse cytoplasmic pattern were compared among the different treatment groups to assess Cyt c redistribution.

In parallel, Western blotting was performed to detect the expression levels of mitochondrial apoptosis-related proteins, including BAX(1:1000, Cat. No. 50599-1-Ig, Proteintech Group, Inc., Wuhan, China), BCL-2(1:1000), cleaved Caspase-9(1:1000, Cat. No. 10380-1-AP, Proteintech Group, Inc., Wuhan, China), and cleaved Caspase-3(1:1000, Cat. No. 68773-1-Ig, Proteintech Group, Inc., Wuhan, China), to comprehensively evaluate activation of the mitochondrial apoptotic pathway.

### 2.15 Mitochondrial fluorescence staining

MitoTracker Red CMXRos and MitoSOX Red (Beyotime Biotechnology, Shanghai, China) were used to evaluate mitochondrial functional status and mitochondrial reactive oxygen species (mtROS) levels, respectively. Cells from the indicated treatment groups were stained according to the manufacturer’s instructions. Fluorescence images were subsequently acquired using a laser scanning confocal microscope. Fluorescence intensity and mitochondrial morphology were compared among the different treatment groups under identical imaging settings (20).

### 2.16 Tumor cell lysate loading of dendritic cells and DC–T cell co-culture

Peripheral blood was collected from healthy volunteers, and peripheral blood mononuclear cells (PBMCs) were isolated by density-gradient centrifugation. Monocytes were enriched by adherence and cultured in RPMI-1640 complete medium supplemented with recombinant human granulocyte-macrophage colony-stimulating factor (GM-CSF; Cat. No. PHC6025, Thermo Fisher Scientific, Waltham, MA, USA) and recombinant human interleukin-4 (IL-4; Cat. No. PHC0041, Thermo Fisher Scientific, Waltham, MA, USA) to induce differentiation into dendritic cells (DCs). Recombinant human TNF-α (Cat. No. PHC3011, Thermo Fisher Scientific, Waltham, MA, USA) was added during the later stage of culture to promote DC maturation.

Meanwhile, T lymphocytes were obtained from autologous PBMCs. The cells were stimulated with phytohemagglutinin (PHA; Cat. No. 10576015, Thermo Fisher Scientific, Waltham, MA, USA) and subsequently expanded in the presence of recombinant human IL-2 (Cat. No. PHC0026, Thermo Fisher Scientific, Waltham, MA, USA) for use in subsequent co-culture experiments.

Pancreatic cancer cells from the different treatment groups were harvested separately, and tumor cell lysates were prepared by repeated freeze–thaw cycles. The lysates were normalized according to either the same initial cell number or total protein concentration. The normalized tumor cell lysates were then added to the DC culture system for antigen loading. After antigen loading, DCs were collected and co-cultured with autologous T lymphocytes at a DC:T-cell ratio of 1:10 to evaluate the ability of DCs loaded with tumor cell lysates from the different treatment groups to induce T-cell immune responses.

### 2.17 Measurement of IFN-γ secretion

DCs loaded with tumor cell lysates from the different treatment groups were co-cultured with autologous T lymphocytes at a ratio of 1:10. Recombinant human IL-2 and recombinant human IL-12 (Cat. No. PHC0124, Thermo Fisher Scientific, Waltham, MA, USA) were added to the co-culture system, and the cells were maintained at 37 °C in a humidified atmosphere containing 5% CO_2_. Culture supernatants were collected at the same time point on days 1, 2, and 3 of co-culture. After centrifugation to remove cells and cellular debris, the supernatants were stored at −20 °C until analysis.

After all samples had been collected, IFN-γ concentrations in the culture supernatants were measured using a Human IFN-γ ELISA Kit (Cat. No. KE00146, Proteintech Group, Inc., Wuhan, China) according to the manufacturer’s instructions. Differences in IFN-γ secretion among the DC–T cell co-culture groups were compared to evaluate the ability of DCs loaded with pancreatic cancer cell lysates from the different treatment groups to induce T-cell immune activation.

### 2.18 Statistical analysis

All experiments were independently repeated at least three times. Data are presented as the mean ± standard error of the mean (SEM). Statistical analyses were performed using GraphPad Prism 8.0 and R software. Comparisons between two groups were conducted using Student’s t-test, whereas comparisons among multiple groups were performed using one-way analysis of variance (ANOVA) followed by Tukey’s multiple-comparison test. Correlations between variables were analyzed using Pearson’s correlation analysis or Spearman’s rank correlation analysis as appropriate. All statistical tests were two-sided, and a P value < 0.05 was considered statistically significant.

## 3 Results

### 3.1 ESCO1 is differentially expressed across cancers and closely associated with immune infiltration and prognosis

To systematically characterize the expression profile of ESCO1 across human cancers, we first compared its expression levels between tumor and corresponding normal tissues across 33 cancer types using data from The Cancer Genome Atlas (TCGA). ESCO1 was significantly upregulated in multiple cancer types, including breast invasive carcinoma (BRCA), cholangiocarcinoma (CHOL), colon adenocarcinoma (COAD), head and neck squamous cell carcinoma (HNSC), kidney renal clear cell carcinoma (KIRC), liver hepatocellular carcinoma (LIHC), lung adenocarcinoma (LUAD), lung squamous cell carcinoma (LUSC), stomach adenocarcinoma (STAD), thyroid carcinoma (THCA), and uterine corpus endometrial carcinoma (UCEC) (**Figure 1A**).

**Figure 1:**
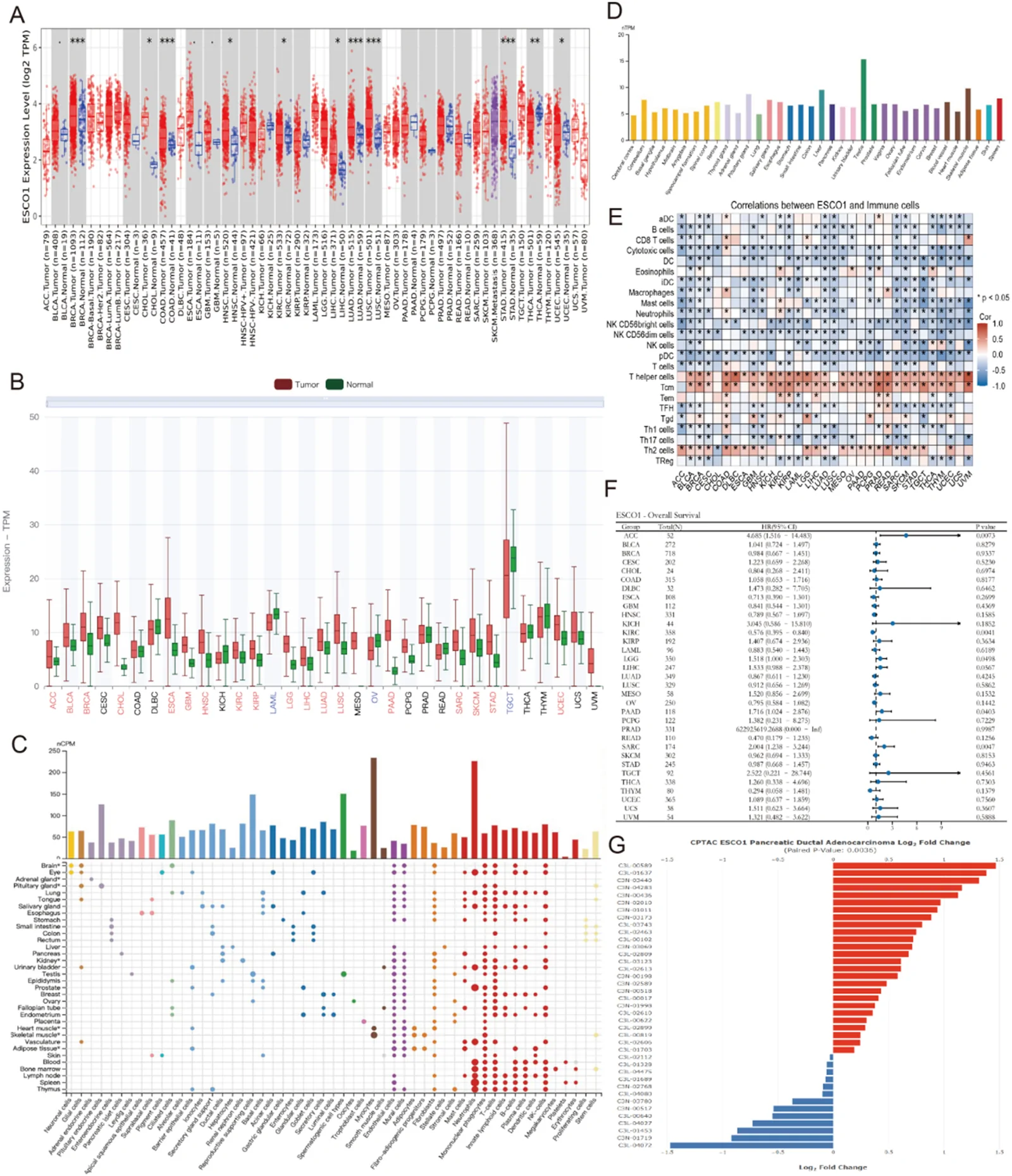
Pan-cancer expression of ESCO1 and its associations with immune infiltration and prognosis. **(A)** ESCO1 expression across 33 tumor types and corresponding normal tissues in TCGA. **(B)** ESCO1 expression across cancers and normal tissues analyzed using GEPIA2. **(C, D)** ESCO1 mRNA expression across different cell types **(C)** and normal human tissues **(D)** in the HPA database. **(E)** Correlations between ESCO1 expression and immune cell infiltration across cancers. **(F)** Prognostic significance of ESCO1 across cancers assessed by Cox regression analysis. **(G)** ESCO1 protein expression in tumor and corresponding adjacent normal tissues in the CPTAC database. *P < 0.05, **P < 0.01, ***P < 0.001, ****P < 0.0001.

Because normal control samples are unavailable or limited for certain cancer types in TCGA, we further validated the expression pattern of ESCO1 using the GEPIA2 platform, which integrates data from TCGA and the Genotype-Tissue Expression (GTEx) project. Consistent with the TCGA-based analysis, ESCO1 showed elevated expression in pancreatic adenocarcinoma (PAAD), adrenocortical carcinoma (ACC), BRCA, CHOL, esophageal carcinoma (ESCA), glioblastoma multiforme (GBM), HNSC, KIRC, kidney renal papillary cell carcinoma (KIRP), brain lower-grade glioma (LGG), LIHC, LUAD, LUSC, sarcoma (SARC), skin cutaneous melanoma (SKCM), STAD, and UCEC (**Figure 1B**). Collectively, these findings demonstrate that ESCO1 is aberrantly upregulated across a broad spectrum of human cancers, suggesting its potential involvement in tumor development and progression.

We next examined the distribution of ESCO1 expression across different cell types and normal human tissues using data from the Human Protein Atlas (HPA). At the single-cell level, ESCO1 was expressed across multiple cell types but exhibited generally low cell-type specificity, with relatively higher expression observed in neutrophils and muscle cells (**Figure 1C**). Similarly, tissue-level analysis revealed widespread ESCO1 expression across normal human tissues, with relatively high expression in the testis but no pronounced tissue specificity overall (**Figure 1D**).

To further investigate the potential relationship between ESCO1 and the tumor immune microenvironment, we analyzed the correlations between ESCO1 expression and immune cell infiltration across multiple cancer types. ESCO1 expression was significantly associated with the infiltration levels of various immune cell populations in multiple cancers (**Figure 1E**), indicating a potential link between ESCO1 expression and the immune landscape of the tumor microenvironment. We subsequently evaluated the prognostic significance of ESCO1 using Cox proportional hazards regression analysis. ESCO1 expression was significantly associated with survival outcomes in several cancer types, including pancreatic cancer (**Figure 1F**).

### 3.2 ESCO1 is highly expressed in pancreatic cancer and shows potential diagnostic value

To further characterize the expression of ESCO1 in pancreatic cancer, we analyzed its expression at the mRNA and protein levels using data from the TCGA and CPTAC databases, respectively. ESCO1 mRNA and protein expression levels were significantly higher in pancreatic cancer tissues than in normal pancreatic tissues (**Figures 2A**, **1G**). To further validate these findings, six paired pancreatic cancer tissues and corresponding adjacent normal tissues were randomly selected, and ESCO1 protein expression was examined by Western blotting. Consistent with the results obtained from the public databases, ESCO1 protein expression was markedly elevated in pancreatic cancer tissues compared with paired adjacent normal tissues (**Figure 2B**).

**Figure 2:**
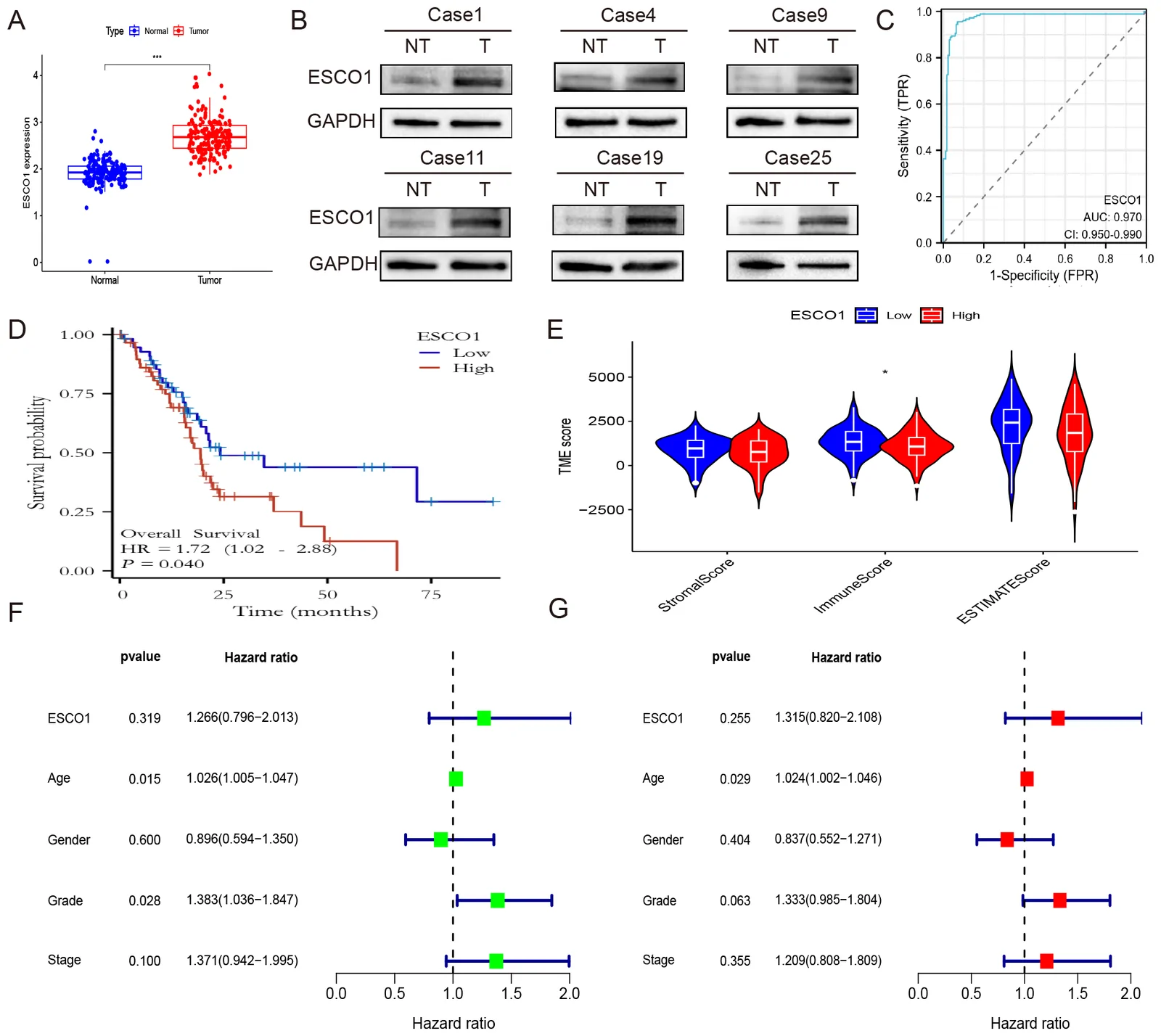
ESCO1 expression, diagnostic value, and prognostic significance in pancreatic cancer. **(A)** ESCO1 expression in pancreatic cancer and normal tissues. **(B)** Western blot analysis of ESCO1 protein expression in six paired pancreatic cancer and adjacent normal tissues. **(C)** ROC curve analysis of the diagnostic performance of ESCO1 in pancreatic cancer. **(D)** Overall survival **(OS)** analysis according to ESCO1 expression. **(E)** Tumor microenvironment characteristics in the high- and low-ESCO1 expression groups. **(F–G)** Univariate **(F)** and multivariate **(G)** Cox regression analyses of the prognostic significance of ESCO1. *P < 0.05, ***P < 0.001.

We next performed receiver operating characteristic (ROC) curve analysis to evaluate the ability of ESCO1 to discriminate pancreatic cancer tissues from normal pancreatic tissues. Sample type (tumor or normal) was used as the classification variable, and ESCO1 expression was used as the predictor variable. The area under the curve (AUC) was calculated to assess its diagnostic performance. ESCO1 effectively distinguished pancreatic cancer tissues from normal pancreatic tissues, suggesting its potential diagnostic value in pancreatic cancer (**Figure 2C**).

### 3.3 High ESCO1 expression is associated with poor prognosis and alterations in the tumor microenvironment in pancreatic cancer

To evaluate the prognostic significance of ESCO1 in pancreatic cancer, transcriptomic expression data and corresponding clinical follow-up information were obtained from the TCGA database. Kaplan–Meier survival analysis was performed to assess the association between ESCO1 expression and overall survival (OS). Patients with high ESCO1 expression had significantly shorter OS than those with low ESCO1 expression, indicating that elevated ESCO1 expression was associated with poor survival outcomes in pancreatic cancer (**Figure 2D**).

Given the significant association between ESCO1 expression and patient survival, we further investigated its relationship with the tumor microenvironment in pancreatic cancer. Patients were stratified into high- and low-ESCO1 expression groups according to the median ESCO1 expression level, and tumor microenvironment scores were compared between the two groups. Significant differences in tumor microenvironment-related scores were observed between the high- and low-ESCO1 expression groups (**Figure 2E**), suggesting that ESCO1 expression was associated with alterations in the tumor microenvironment of pancreatic cancer.

Finally, univariate and multivariate Cox proportional hazards regression analyses were performed to evaluate whether ESCO1 could serve as an independent prognostic factor in pancreatic cancer. Neither univariate nor multivariate analysis revealed a statistically significant association between ESCO1 expression and OS (**Figures 2F, G**), indicating that ESCO1 was not an independent prognostic factor for OS in patients with pancreatic cancer.

### 3.4 NUFIP2 is positively correlated with ESCO1 expression and associated with poor prognosis in pancreatic cancer

To identify potential molecules associated with ESCO1 in pancreatic cancer, Pearson correlation analysis was performed using pancreatic cancer transcriptomic data. ESCO1 expression was significantly correlated with the expression of multiple genes, among which MIB1, NUFIP2, ZNF12, ZMYM2, BRWD3, SF3B1, VKORC1, SIL1, CHID1, POLR2L, and PRDX4 showed relatively strong correlations with ESCO1 (**Figures 3A, B**).

**Figure 3:**
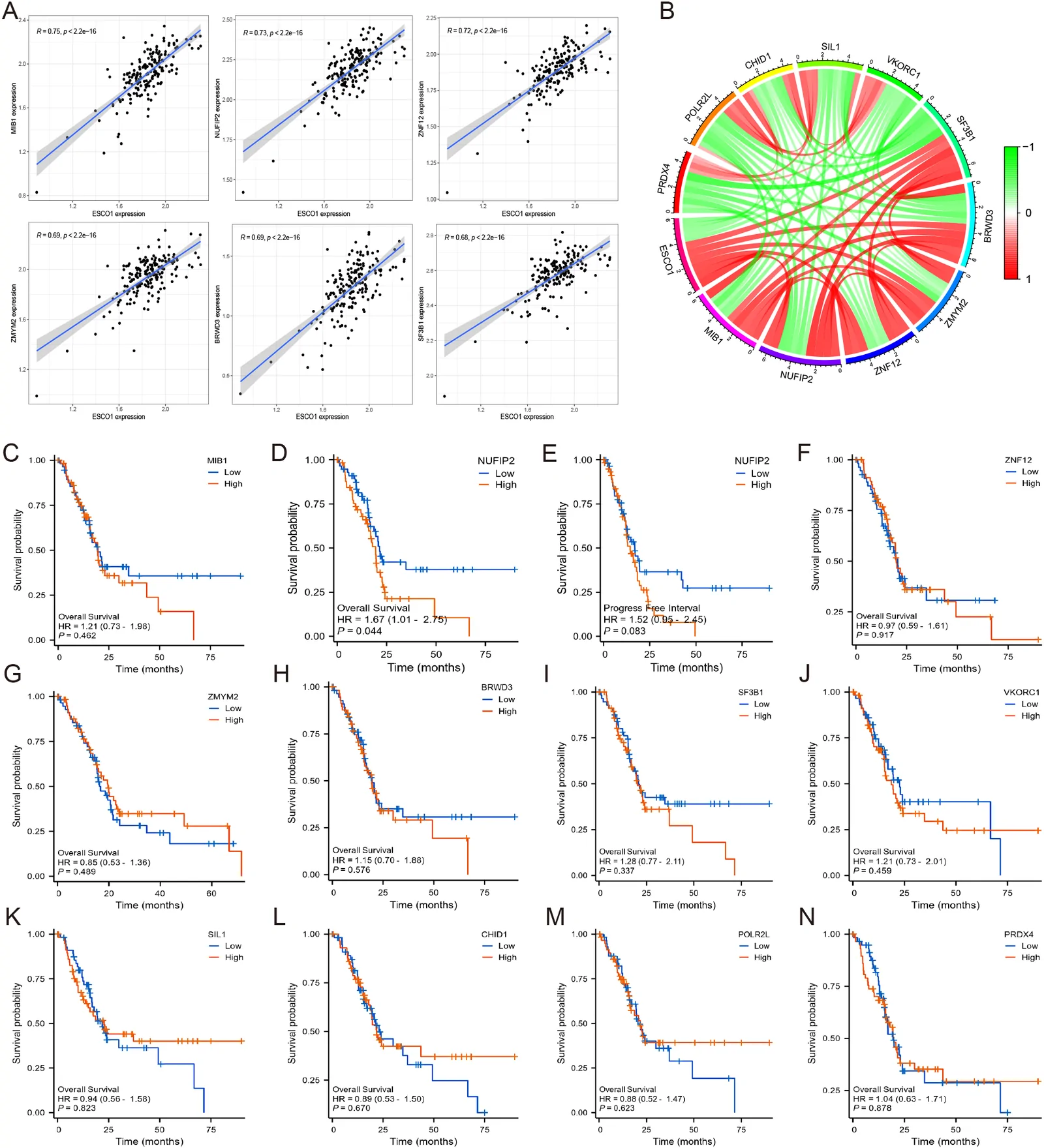
Screening and prognostic analysis of ESCO1-correlated genes. **(A)** Pearson correlation analysis identifying candidate genes significantly correlated with ESCO1 expression. **(B)** Expression correlations between ESCO1 and representative candidate genes. **(C–N)** Kaplan–Meier survival analyses evaluating the associations between candidate gene expression and overall survival (OS) in patients with pancreatic cancer.

To further assess the potential clinical relevance of these ESCO1-correlated genes, we evaluated the associations between their expression levels and overall survival (OS) in patients with pancreatic cancer. Kaplan–Meier survival analysis revealed no significant associations between OS and the expression of MIB1, ZNF12, ZMYM2, BRWD3, SF3B1, VKORC1, SIL1, CHID1, POLR2L, or PRDX4. In contrast, high NUFIP2 expression was significantly associated with shorter OS in patients with pancreatic cancer (**Figures 3C–N**).

Given its significant positive correlation with ESCO1 expression and its association with poor survival, NUFIP2 was identified as a candidate molecule potentially related to ESCO1-mediated pancreatic cancer progression. Accordingly, NUFIP2 was selected for subsequent functional and mechanistic investigations.

### 3.5 NUFIP2 is highly expressed in pancreatic cancer and shows potential diagnostic value

Using data from the TCGA database, we first analyzed NUFIP2 expression in pancreatic cancer and normal pancreatic tissues. NUFIP2 expression was significantly higher in pancreatic cancer tissues than in normal pancreatic tissues (**Figure 4A**).

**Figure 4:**
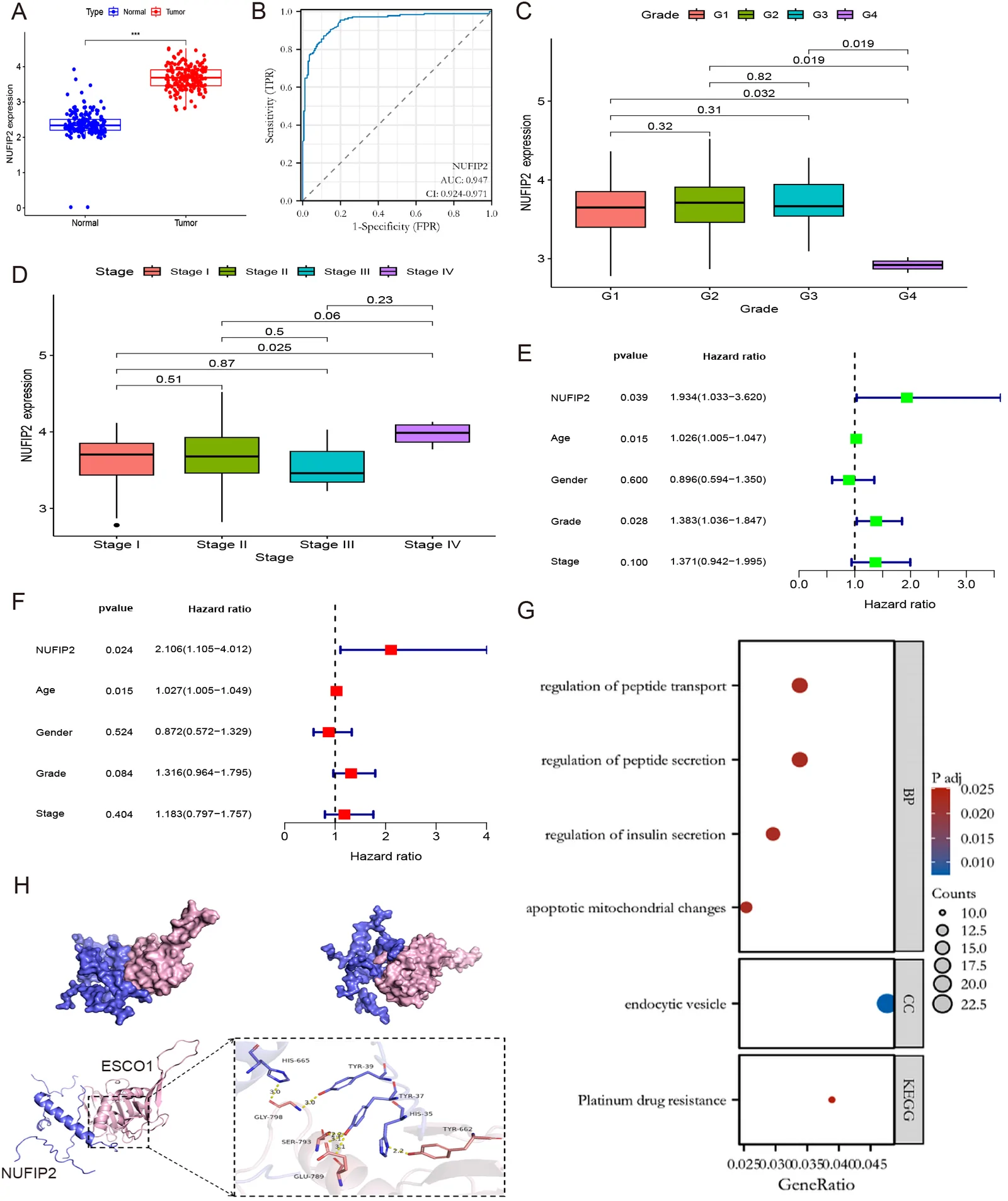
NUFIP2 expression, clinical significance, and functional analysis in pancreatic cancer. **(A)** Differential expression of NUFIP2 between pancreatic cancer and normal tissues. **(B)** ROC curve analysis of the diagnostic performance of NUFIP2 in pancreatic cancer. **(C, D)** Differential NUFIP2 expression across histological grades **(C)** and tumor stages **(D)**. **(E, F)** Univariate **(E)** and multivariate **(F)** Cox regression analyses of the prognostic significance of NUFIP2. **(G)** GO and KEGG enrichment analyses of NUFIP2-related differentially expressed genes. **(H)** Molecular docking model of the potential protein–protein interaction between ESCO1 and NUFIP2. ***P < 0.001.

To further evaluate the ability of NUFIP2 to discriminate pancreatic cancer tissues from normal pancreatic tissues, receiver operating characteristic (ROC) curve analysis was performed. Sample type (tumor or normal) was used as the classification variable, and NUFIP2 expression was used as the predictor variable. The area under the curve (AUC) was calculated to assess its diagnostic performance. NUFIP2 showed a high AUC value and effectively distinguished pancreatic cancer tissues from normal pancreatic tissues, suggesting its potential diagnostic value in pancreatic cancer (**Figure 4B**).

### 3.6 NUFIP2 expression is associated with clinicopathological features and has independent prognostic value in pancreatic cancer

To further investigate the association between NUFIP2 expression and clinicopathological characteristics in pancreatic cancer, we compared NUFIP2 expression levels across different histological grades and tumor stages. Group comparisons were performed using the Wilcoxon rank-sum test or Kruskal–Wallis test, followed by Dunn’s *post hoc* test for multiple comparisons. NUFIP2 expression differed significantly across histological grades and tumor stages. Specifically, NUFIP2 expression was lower in G4 tumors than in G1, G2, and G3 tumors (**Figure 4C**). In addition, a significant difference in NUFIP2 expression was observed between stage IV and stage I tumors (**Figure 4D**). Notably, the relatively low expression of NUFIP2 in G4 tumors may reflect differences in the biological characteristics associated with histological grade, the limited sample size of the G4 subgroup, and the molecular heterogeneity of pancreatic cancer.

We subsequently performed univariate and multivariate Cox proportional hazards regression analyses to evaluate the prognostic significance of NUFIP2 for overall survival (OS) in patients with pancreatic cancer. NUFIP2 expression was significantly associated with OS in both univariate and multivariate analyses, indicating that NUFIP2 may serve as an independent prognostic factor for OS in patients with pancreatic cancer (**Figures 4E, F**).

### 3.7 GO and KEGG enrichment analyses of NUFIP2-related differentially expressed genes

To further explore the potential biological functions and signaling pathways associated with NUFIP2-related differentially expressed genes, Gene Ontology (GO) and Kyoto Encyclopedia of Genes and Genomes (KEGG) enrichment analyses were performed using the clusterProfiler package. NUFIP2-related differentially expressed genes were predominantly enriched in biological processes associated with secretion and transport, including regulation of peptide transport, regulation of peptide secretion, and regulation of insulin secretion. In addition, these genes were associated with apoptosis-related mitochondrial changes and were enriched in cellular components such as endocytic vesicles. KEGG analysis further revealed enrichment in cancer-related pathways, including platinum drug resistance (**Figure 4G**).

### 3.8 Molecular docking analysis of the potential interaction between ESCO1 and NUFIP2

To investigate the potential protein–protein interaction between ESCO1 and NUFIP2, molecular docking analysis was performed using the HDOCK platform. Representative docking models with favorable predicted binding stability were selected based on the docking scores and predicted binding conformations and subsequently visualized in three dimensions using PyMOL.

Molecular docking analysis revealed that ESCO1 and NUFIP2 could form a relatively stable predicted binding conformation, with potential intermolecular interactions at the predicted protein–protein interface (**Figure 4H**). These findings suggest a potential protein–protein interaction between ESCO1 and NUFIP2 and provide a rationale for subsequent experimental validation using co-immunoprecipitation and confocal immunofluorescence analyses.

### 3.9 Single-cell expression patterns of ESCO1 and NUFIP2 and cell–cell communication in pancreatic cancer

To further characterize the cellular distribution of ESCO1 and NUFIP2 within the pancreatic cancer microenvironment, we analyzed their expression patterns using single-cell RNA sequencing data. UMAP-based dimensionality reduction and clustering identified 12 major cell populations, including B cells, CD4^+^ T cells, CD8^+^ T cells, NK cells, macrophages, monocytes, neutrophils, epithelial cells, hepatocytes, fibroblasts, endothelial cells, and stem cell populations (**Figure 5A**). Cell–cell communication analysis revealed extensive signaling interactions among these cell populations, with epithelial cells, endothelial cells, T cells, and fibroblasts showing prominent intercellular communication (**Figures 5B, C**). These findings highlight the complex intercellular communication network within the pancreatic cancer microenvironment.

**Figure 5:**
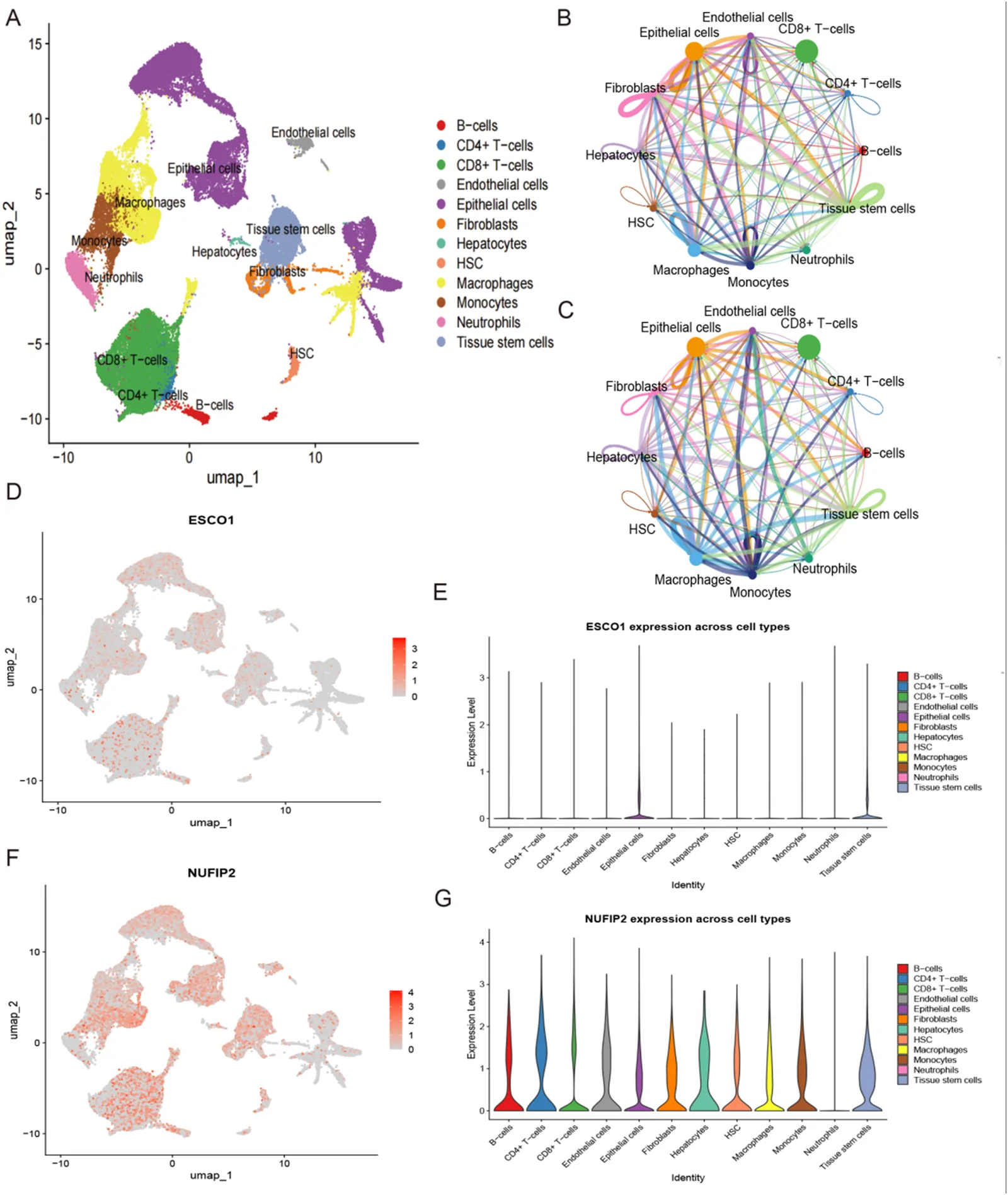
Single-cell expression of ESCO1 and NUFIP2 and cell–cell communication in pancreatic cancer. **(A)** UMAP analysis identified 12 major cell populations in pancreatic cancer. **(B, C)** Cell–cell communication networks among different cell populations in the pancreatic cancer microenvironment. **(D, E)** Expression patterns of ESCO1 across different cell populations. **(F, G)** Expression patterns of NUFIP2 across different cell populations.

We next examined the single-cell expression patterns of ESCO1 and NUFIP2. ESCO1 was predominantly expressed in epithelial cells, with moderate expression also detected in endothelial cells, whereas its expression was relatively low in most other cell populations (**Figures 5D, E**). Similarly, NUFIP2 was prominently expressed in epithelial cells, with detectable expression also observed in several immune and other cell populations (**Figures 5F, G**).

### 3.10 ESCO1 promotes the malignant phenotype of pancreatic cancer cells

To characterize ESCO1 expression in pancreatic cancer cells, Western blotting was performed in four pancreatic cancer cell lines (PANC-1, SW1990, ASPC-1, and MIAPaCa-2) and the normal pancreatic ductal epithelial cell line HPDE6-C7. ESCO1 protein expression was significantly higher in multiple pancreatic cancer cell lines than in HPDE6-C7 cells (**Figure 6A**).

**Figure 6:**
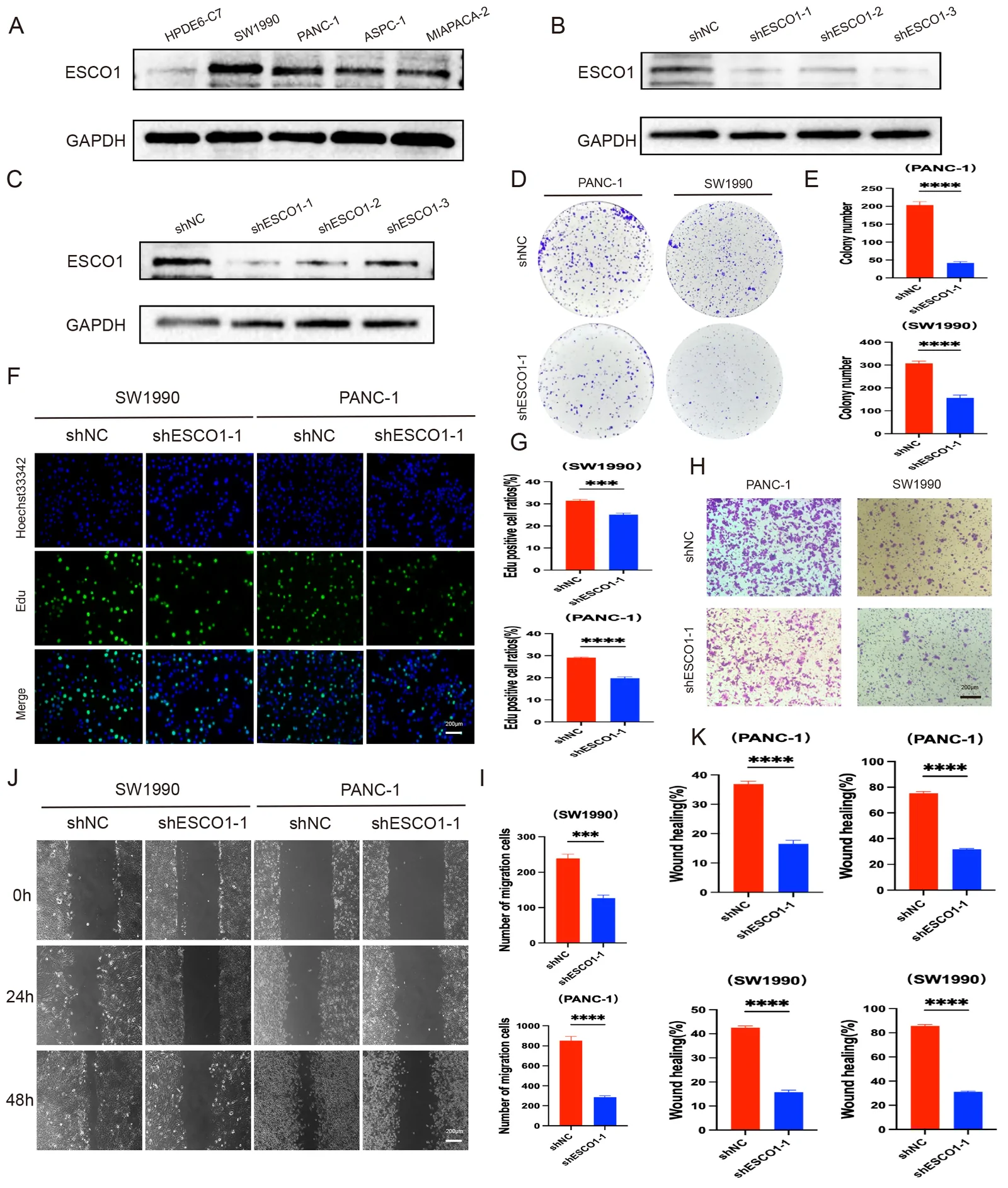
ESCO1 knockdown suppresses pancreatic cancer cell proliferation and migration. **(A)** ESCO1 protein expression in HPDE6-C7 and pancreatic cancer cell lines. **(B, C)** Western blot validation of ESCO1 knockdown in PANC-1 and SW1990 cells. **(D, E)** Colony formation assays following ESCO1 knockdown. **(F, G)** EdU assays assessing cell proliferation following ESCO1 knockdown. **(H, I)** Transwell assays assessing cell migration following ESCO1 knockdown. **(J, K)** Wound-healing assays assessing cell migration following ESCO1 knockdown. ***P < 0.001, ****P < 0.0001.

PANC-1 and SW1990 cells, which exhibited relatively high endogenous ESCO1 expression, were selected for subsequent loss-of-function experiments. ESCO1 was stably knocked down using lentiviral shRNAs, and Western blotting confirmed a marked reduction in ESCO1 protein expression in both cell lines (**Figures 6B, C**), confirming efficient ESCO1 knockdown.

We next examined the effects of ESCO1 knockdown on the malignant phenotype of pancreatic cancer cells. Colony formation and EdU incorporation assays demonstrated that ESCO1 knockdown significantly reduced colony-forming capacity and cell proliferation (**Figures 6D–G**). Transwell migration and wound-healing assays further showed that ESCO1 knockdown markedly impaired the migratory capacity of pancreatic cancer cells (**Figures 6H–K**). In addition, Annexin V-FITC/PI flow cytometric analysis revealed a significant increase in the proportion of apoptotic cells following ESCO1 knockdown (**Figures 7A, B**).

**Figure 7:**
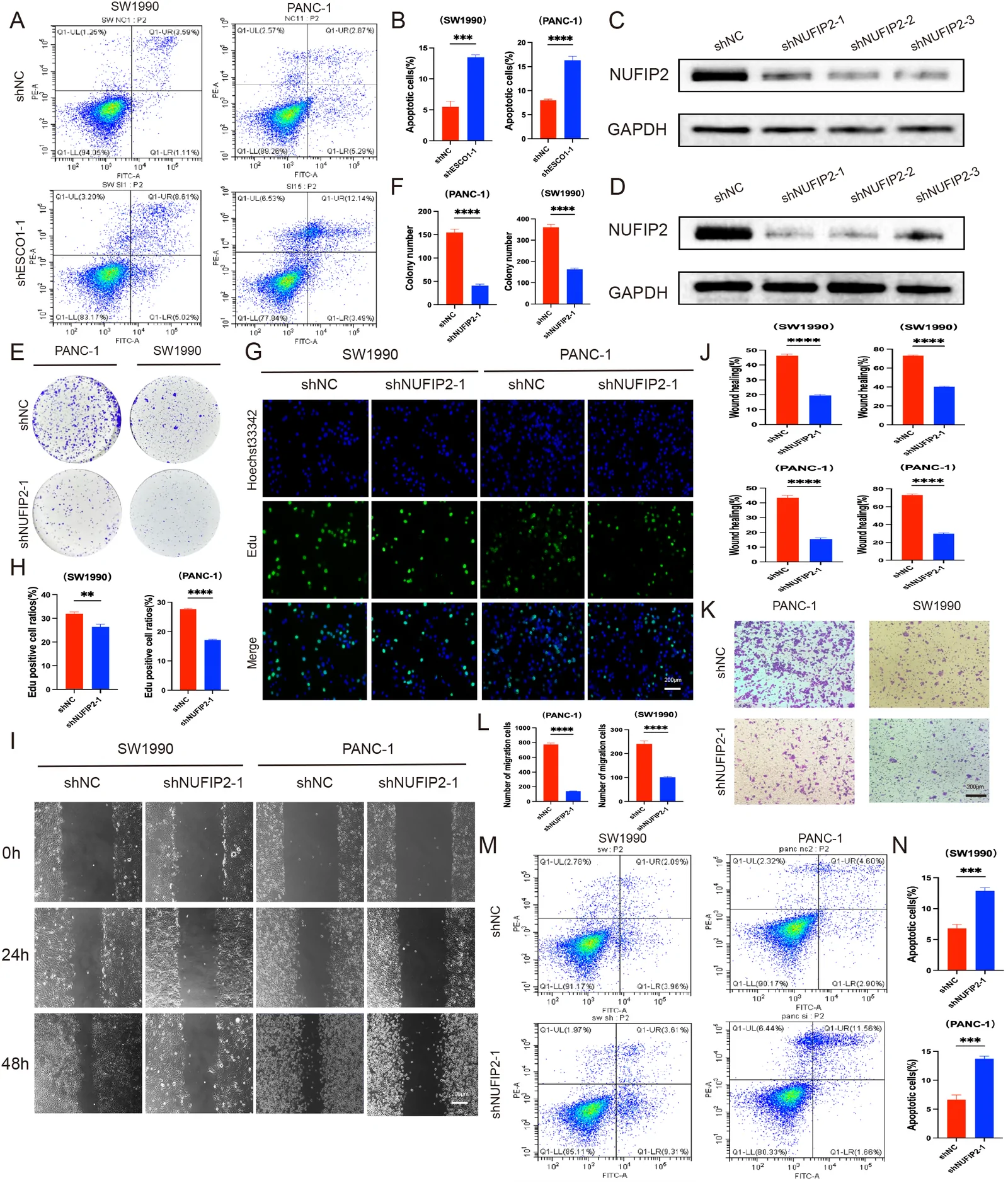
ESCO1 and NUFIP2 knockdown suppress malignant phenotypes of pancreatic cancer cells. **(A, B)** Flow cytometric analysis of apoptosis following ESCO1 knockdown. **(C, D)** Western blot validation of NUFIP2 knockdown in PANC-1 and SW1990 cells. **(E, F)** Colony formation assays following NUFIP2 knockdown. **(G, H)** EdU assays assessing cell proliferation following NUFIP2 knockdown. **(I, J)** Transwell assays assessing cell migration following NUFIP2 knockdown. **(K, L)** Wound-healing assays assessing cell migration following NUFIP2 knockdown. **(M, N)** Flow cytometric analysis of apoptosis following NUFIP2 knockdown. **P < 0.01, ***P < 0.001, ****P < 0.0001.

### 3.11 NUFIP2 promotes the malignant phenotype of pancreatic cancer cells

To investigate the functional role of NUFIP2 in pancreatic cancer cells, stable NUFIP2-knockdown cell lines were established using lentivirus-mediated shRNA interference. Western blotting confirmed efficient NUFIP2 knockdown in PANC-1 and SW1990 cells (**Figures 7C, D**).

We next examined the effects of NUFIP2 knockdown on pancreatic cancer cell proliferation. Colony formation and EdU incorporation assays demonstrated that NUFIP2 knockdown significantly reduced colony-forming capacity and cell proliferation (**Figures 7E–H**). Transwell migration and wound-healing assays further showed that NUFIP2 knockdown markedly impaired the migratory capacity of pancreatic cancer cells (**Figures 7I–L**). In addition, Annexin V-FITC/PI flow cytometric analysis revealed a significant increase in the proportion of apoptotic cells following NUFIP2 knockdown (**Figures 7M, N**).

### 3.12 ESCO1 promotes the malignant phenotype and tumor growth of pancreatic cancer through NUFIP2

To elucidate the regulatory relationship between ESCO1 and NUFIP2, we manipulated the expression of either ESCO1 or NUFIP2 in PANC-1 and SW1990 cells and examined the expression of the other protein. Western blotting showed that modulation of ESCO1 expression resulted in corresponding changes in NUFIP2 protein levels (**Figures 8A, B**), whereas modulation of NUFIP2 expression had no appreciable effect on ESCO1 protein levels (**Figures 8C, D**), suggesting that NUFIP2 may function downstream of ESCO1.

**Figure 8:**
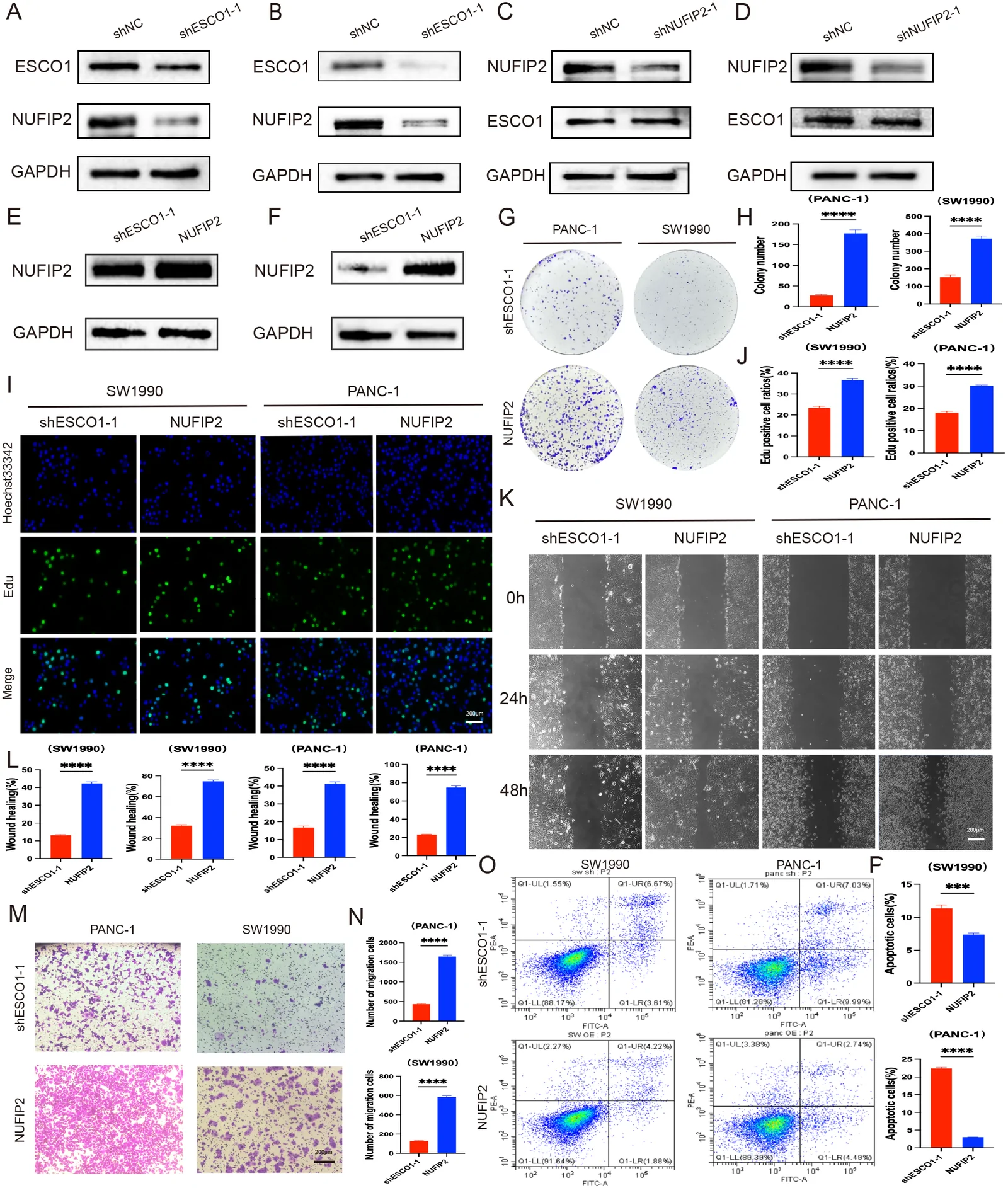
NUFIP2 mediates the effects of ESCO1 on the malignant phenotype of pancreatic cancer cells. **(A, B)** NUFIP2 protein expression following ESCO1 knockdown or overexpression. **(C, D)** ESCO1 protein expression following modulation of NUFIP2 expression. **(E, F)** Western blot validation of NUFIP2 overexpression in ESCO1-knockdown cells. **(G, H)** Colony formation assays assessing the rescue effect of NUFIP2 overexpression. **(I, J)** EdU assays assessing the rescue of cell proliferation by NUFIP2 overexpression. **(K, L)** Transwell assays assessing the rescue of cell migration by NUFIP2 overexpression. **(M, N)** Wound-healing assays assessing the rescue of cell migration by NUFIP2 overexpression. **(O, P)** Flow cytometric analysis of apoptosis following NUFIP2 overexpression in ESCO1-knockdown cells. ***P < 0.001, ****P < 0.0001.

To further determine whether NUFIP2 mediates the effects of ESCO1 on the malignant phenotype of pancreatic cancer cells, NUFIP2 expression was restored in ESCO1-knockdown cells, and successful NUFIP2 overexpression was confirmed by Western blotting (**Figures 8E, F**). Functional assays demonstrated that restoration of NUFIP2 expression significantly rescued the reductions in colony-forming capacity and cell proliferation induced by ESCO1 knockdown (**Figures 8G–J**) and markedly restored cell migration (**Figures 8K–N**). Furthermore, flow cytometric analysis showed that NUFIP2 overexpression significantly attenuated ESCO1 knockdown-induced apoptosis (**Figures 8O, P**). These findings indicate that NUFIP2 plays an important role in mediating the effects of ESCO1 on pancreatic cancer cell proliferation, migration, and apoptosis.

We subsequently used a xenograft model to further validate the functional role of the ESCO1–NUFIP2 axis *in vivo*. Knockdown of either ESCO1 or NUFIP2 significantly suppressed tumor growth, whereas restoration of NUFIP2 expression partially reversed the tumor growth inhibition induced by ESCO1 knockdown (**Figure 9A**). Consistent with these findings, immunohistochemical staining of xenograft tumors revealed reduced Ki-67 and BCL-2 expression following ESCO1 or NUFIP2 knockdown, whereas restoration of NUFIP2 expression partially reversed these changes (**Figure 9B**). These results further support the role of NUFIP2 in mediating the effects of ESCO1 on tumor cell proliferation, apoptosis, and tumor growth *in vivo*.

**Figure 9:**
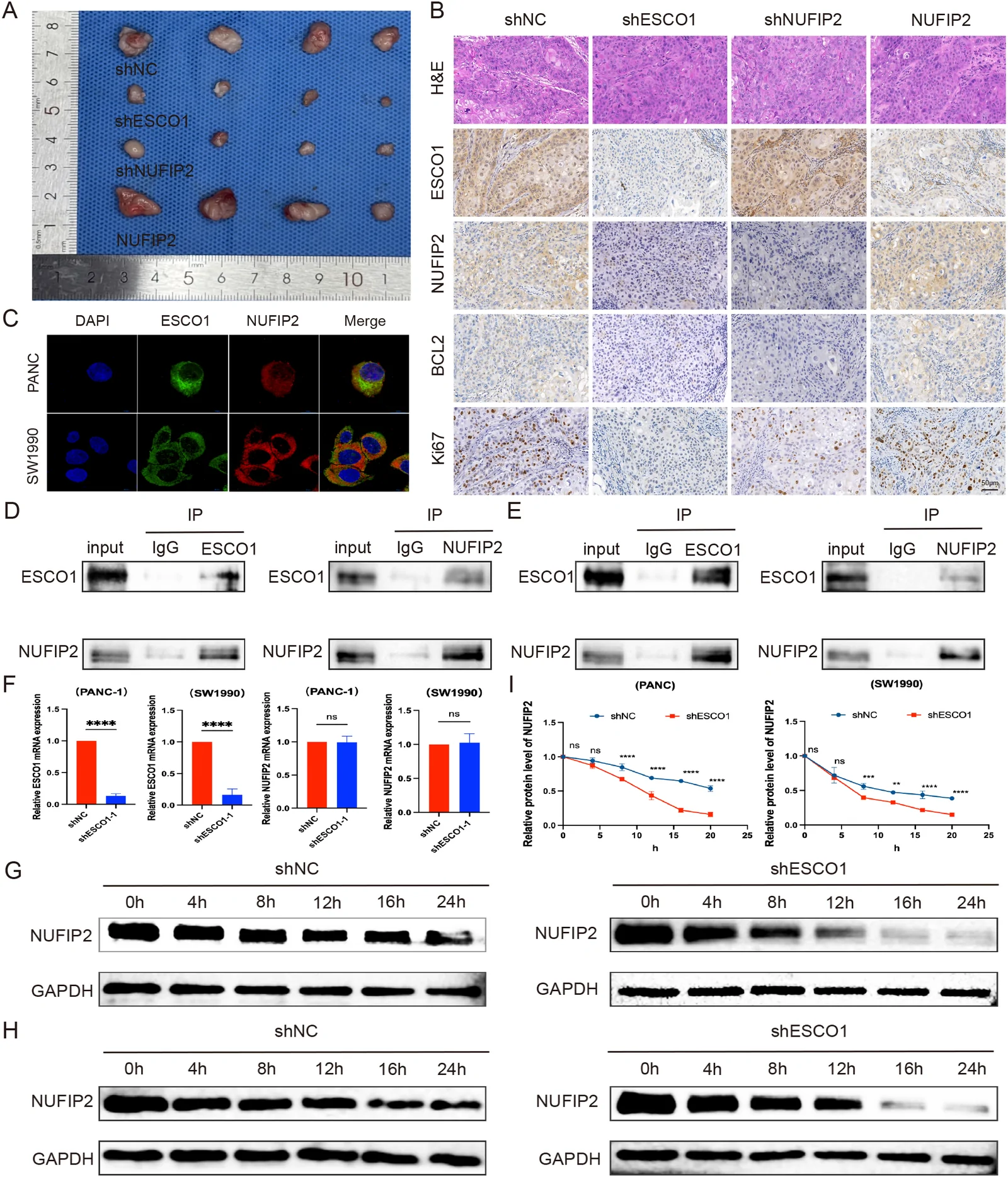
NUFIP2 mediates ESCO1-driven tumor growth, and ESCO1 interacts with NUFIP2 to maintain its protein stability. **(A)** Xenograft tumor growth following ESCO1 knockdown, NUFIP2 knockdown, or NUFIP2 restoration. **(B)** Immunohistochemical staining of Ki-67 and BCL-2 in xenograft tumors. **(C)** Confocal immunofluorescence analysis of ESCO1 and NUFIP2 colocalization. **(D, E)** Co-IP analysis of the interaction between ESCO1 and NUFIP2 in PANC-1 and SW1990 cells. **(F)** NUFIP2 mRNA expression following ESCO1 knockdown. **(G–I)** CHX chase analysis of NUFIP2 protein stability in PANC-1 and SW1990 cells. **P < 0.01, ***P < 0.001,****P < 0.0001.

### 3.13 ESCO1 interacts with NUFIP2 and maintains NUFIP2 protein stability

To investigate the potential interaction between ESCO1 and NUFIP2 in pancreatic cancer cells, we first examined their subcellular localization by confocal immunofluorescence microscopy. ESCO1 and NUFIP2 exhibited substantial intracellular colocalization (**Figure 9C**), indicating spatial proximity between the two proteins and providing initial evidence for a potential interaction.

We next performed co-immunoprecipitation (Co-IP) assays to further examine the association between ESCO1 and NUFIP2. Reciprocal Co-IP experiments demonstrated that NUFIP2 was detected in ESCO1 immunoprecipitates and, conversely, ESCO1 was detected in NUFIP2 immunoprecipitates. Consistent results were obtained in both PANC-1 and SW1990 cells (**Figures 9D, E**), supporting an association between ESCO1 and NUFIP2 in pancreatic cancer cells.

To determine whether ESCO1 regulates NUFIP2 at the transcriptional level, NUFIP2 mRNA expression was examined following ESCO1 knockdown. qPCR analysis showed that ESCO1 knockdown did not significantly alter NUFIP2 mRNA levels (**Figure 9F**), suggesting that the regulation of NUFIP2 by ESCO1 is unlikely to occur primarily at the transcriptional level and may instead involve post-transcriptional or post-translational mechanisms, including regulation of protein stability.

Based on these findings, we further assessed NUFIP2 protein stability using a cycloheximide (CHX) chase assay. ESCO1-knockdown PANC-1 and SW1990 cells were treated with CHX, and NUFIP2 protein levels were monitored at different time points. Compared with the corresponding control cells, ESCO1 knockdown markedly accelerated NUFIP2 protein degradation and significantly shortened its protein half-life (**Figures 9G–I**), indicating that ESCO1 contributes to the maintenance of NUFIP2 protein stability.

### 3.14 ESCO1 maintains NUFIP2 protein stability by inhibiting K48-linked ubiquitination

To further elucidate the molecular mechanism by which ESCO1 maintains NUFIP2 protein stability, ESCO1, NUFIP2, and HA-tagged ubiquitin (HA-Ub) were co-expressed in 293T cells, and NUFIP2 ubiquitination was assessed by co-immunoprecipitation. NUFIP2 exhibited robust ubiquitination, whereas ESCO1 overexpression markedly reduced its ubiquitination level (**Figure 10A**), suggesting that ESCO1 may maintain NUFIP2 protein stability by suppressing its ubiquitination.

**Figure 10:**
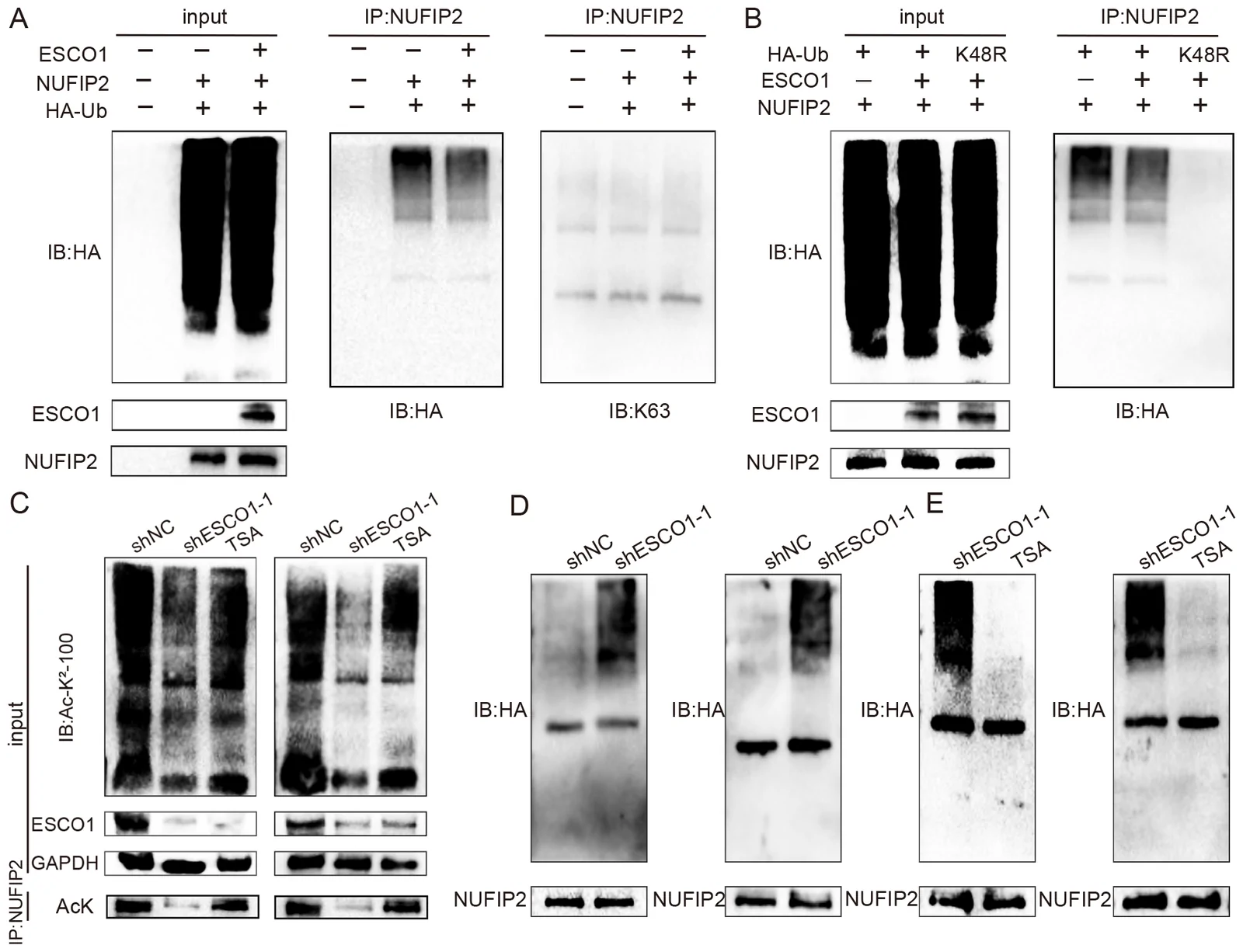
ESCO1 maintains NUFIP2 protein stability by regulating its acetylation and K48-linked ubiquitination. **(A)** NUFIP2 ubiquitination following ESCO1 overexpression in 293T cells. **(B)** Analysis of K48-linked ubiquitination of NUFIP2 using a K48-mutant ubiquitin construct. **(C)** NUFIP2 acetylation following ESCO1 knockdown and TSA treatment in PANC-1 and SW1990 cells. **(D)** NUFIP2 ubiquitination following ESCO1 knockdown in PANC-1 and SW1990 cells. **(E)** NUFIP2 ubiquitination following TSA treatment in ESCO1-knockdown PANC-1 and SW1990 cells.

To further determine the type of ubiquitin linkage involved in NUFIP2 ubiquitination, a ubiquitin construct carrying a K48 mutation was used. Compared with wild-type ubiquitin, mutation of the K48 residue markedly reduced the ubiquitination signal of NUFIP2 (**Figure 10B**), indicating that NUFIP2 ubiquitination predominantly involves K48-linked ubiquitin chains. Given that K48-linked polyubiquitination is commonly associated with proteasomal degradation, these findings suggest that NUFIP2 may undergo degradation through a K48-linked ubiquitination pathway.

Given the acetyltransferase activity of ESCO1, we next examined whether ESCO1 regulates NUFIP2 acetylation. In PANC-1 and SW1990 cells, ESCO1 knockdown markedly reduced NUFIP2 acetylation, whereas treatment with the histone deacetylase inhibitor trichostatin A (TSA) partially restored its acetylation level (**Figure 10C**), suggesting that ESCO1 contributes to the regulation of NUFIP2 acetylation.

Further ubiquitination assays showed that ESCO1 knockdown markedly increased NUFIP2 ubiquitination, whereas TSA treatment partially attenuated this increase (**Figures 10D, E**). Collectively, these findings suggest that ESCO1 promotes NUFIP2 acetylation and suppresses its K48-linked ubiquitination, thereby reducing NUFIP2 degradation and maintaining its protein stability.

### 3.15 ESCO1 maintains mitochondrial function and suppresses mitochondrial apoptosis through acetylation-mediated regulation of NUFIP2

To further investigate the role of the ESCO1/NUFIP2 axis and acetylation-dependent regulation in mitochondrial function and apoptosis, we first examined the expression of the mitochondrial apoptosis-related proteins BCL2, BAX, cleaved caspase-3 (c-Caspase-3), and cleaved caspase-9 (c-Caspase-9). Western blotting showed that ESCO1 knockdown markedly decreased BCL2 expression while significantly increasing the levels of BAX, c-Caspase-9, and c-Caspase-3 in PANC-1 and SW1990 cells. Notably, NUFIP2 overexpression partially reversed these changes (**Figures 11A–D**), suggesting that NUFIP2 mediates, at least in part, the regulatory effect of ESCO1 on mitochondrial apoptosis.

**Figure 11:**
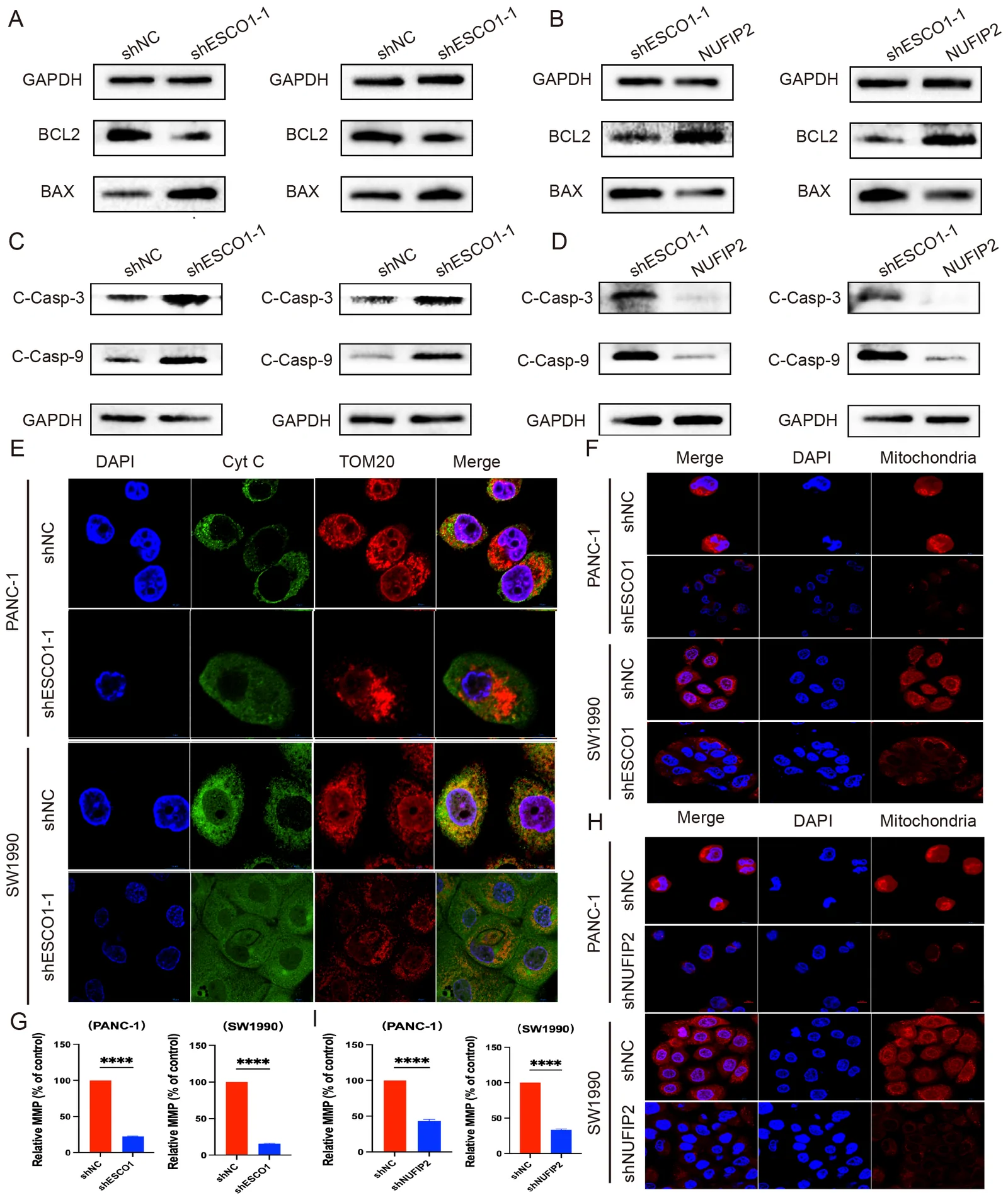
ESCO1 regulates mitochondrial apoptosis through NUFIP2. **(A–D)** Western blot analysis of BCL2, BAX, cleaved caspase-9, and cleaved caspase-3 following ESCO1 knockdown and NUFIP2 overexpression in PANC-1 and SW1990 cells. **(E)** Confocal immunofluorescence showing the subcellular distribution of cytochrome c and its colocalization with TOM20 after ESCO1 knockdown. **(F–H)** MitoTracker Red CMXRos staining and quantification of mitochondrial membrane potential following ESCO1 or NUFIP2 knockdown. ****P < 0.0001.

We next examined the subcellular localization of cytochrome c (Cyt c) by confocal immunofluorescence microscopy. In control cells, Cyt c exhibited a predominantly punctate distribution and showed extensive colocalization with the mitochondrial marker TOM20. Following ESCO1 knockdown, the punctate Cyt c signal was markedly reduced and replaced by a diffuse cytoplasmic distribution in both PANC-1 and SW1990 cells, indicating the release of Cyt c from mitochondria into the cytoplasm (**Figure 11E**). These findings further suggest that ESCO1 depletion promotes Cyt c release and activation of the mitochondrial apoptotic pathway.

Mitochondrial membrane potential was subsequently assessed using MitoTracker Red CMXRos staining. Knockdown of either ESCO1 or NUFIP2 significantly reduced MitoTracker Red CMXRos fluorescence intensity in PANC-1 and SW1990 cells, indicating mitochondrial membrane depolarization and impaired mitochondrial function (**Figures 11F–H**). Notably, treatment with the histone deacetylase inhibitor trichostatin A (TSA) partially restored the reduction in MitoTracker Red CMXRos fluorescence induced by ESCO1 knockdown (**Figures 12A, B**), suggesting that restoration of acetylation levels can partially alleviate mitochondrial dysfunction caused by ESCO1 depletion. These findings further support a role for ESCO1-mediated acetylation in maintaining mitochondrial function.

**Figure 12:**
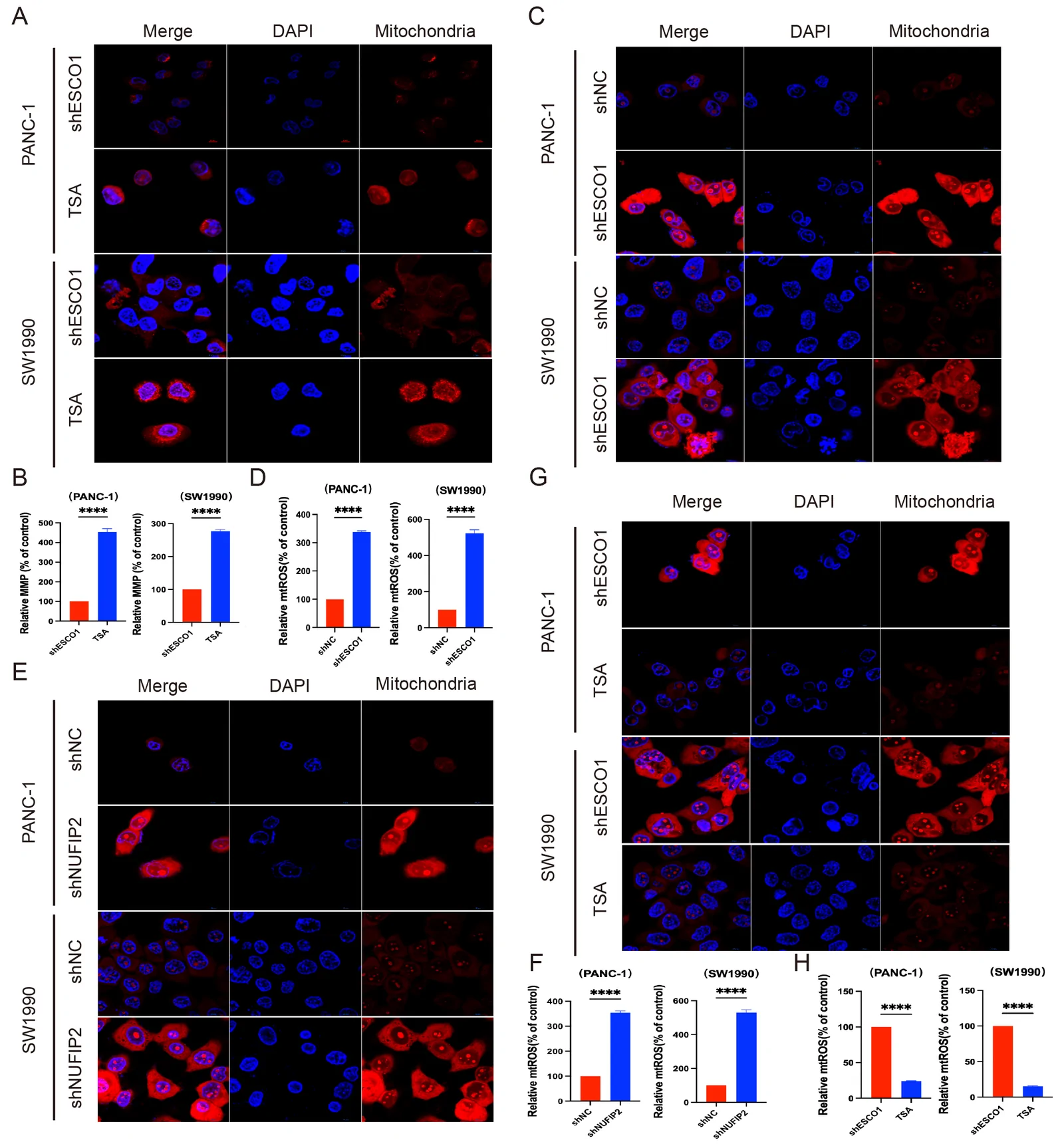
Acetylation-dependent regulation of mitochondrial function by ESCO1. **(A, B)** MitoTracker Red CMXRos staining and quantification after ESCO1 knockdown with or without TSA treatment. **(C–F)** MitoSOX Red staining and quantification of mitochondrial superoxide levels following ESCO1 or NUFIP2 knockdown. **(G, H)** MitoSOX Red staining and quantification after ESCO1 knockdown with or without TSA treatment. ****P < 0.0001.

Mitochondrial superoxide levels were further assessed using MitoSOX Red staining. Knockdown of either ESCO1 or NUFIP2 significantly increased MitoSOX Red fluorescence intensity in PANC-1 and SW1990 cells, indicating increased mitochondrial superoxide production and accumulation of mitochondrial reactive oxygen species (mtROS) (**Figures 12C–F**). Consistently, TSA treatment partially attenuated the increase in MitoSOX Red fluorescence induced by ESCO1 knockdown (**Figures 12G, H**), suggesting that restoration of acetylation levels partially alleviates mtROS accumulation caused by ESCO1 depletion.

### 3.16 The ESCO1/NUFIP2 axis modulates pancreatic cancer–associated antitumor immune responses

To further investigate the relationship between the ESCO1/NUFIP2 axis and the immune microenvironment of pancreatic cancer, we first analyzed the correlations between ESCO1 and NUFIP2 expression and tumor-infiltrating immune cells. Both ESCO1 and NUFIP2 expression were positively correlated with resting memory CD4^+^ T-cell infiltration but negatively correlated with activated NK cells and follicular helper T cells. In addition, ESCO1 expression was significantly negatively correlated with CD8^+^ T-cell infiltration (**Figures 13A, B**). These findings suggest that the ESCO1/NUFIP2 axis is closely associated with the immune landscape of pancreatic cancer.

**Figure 13:**
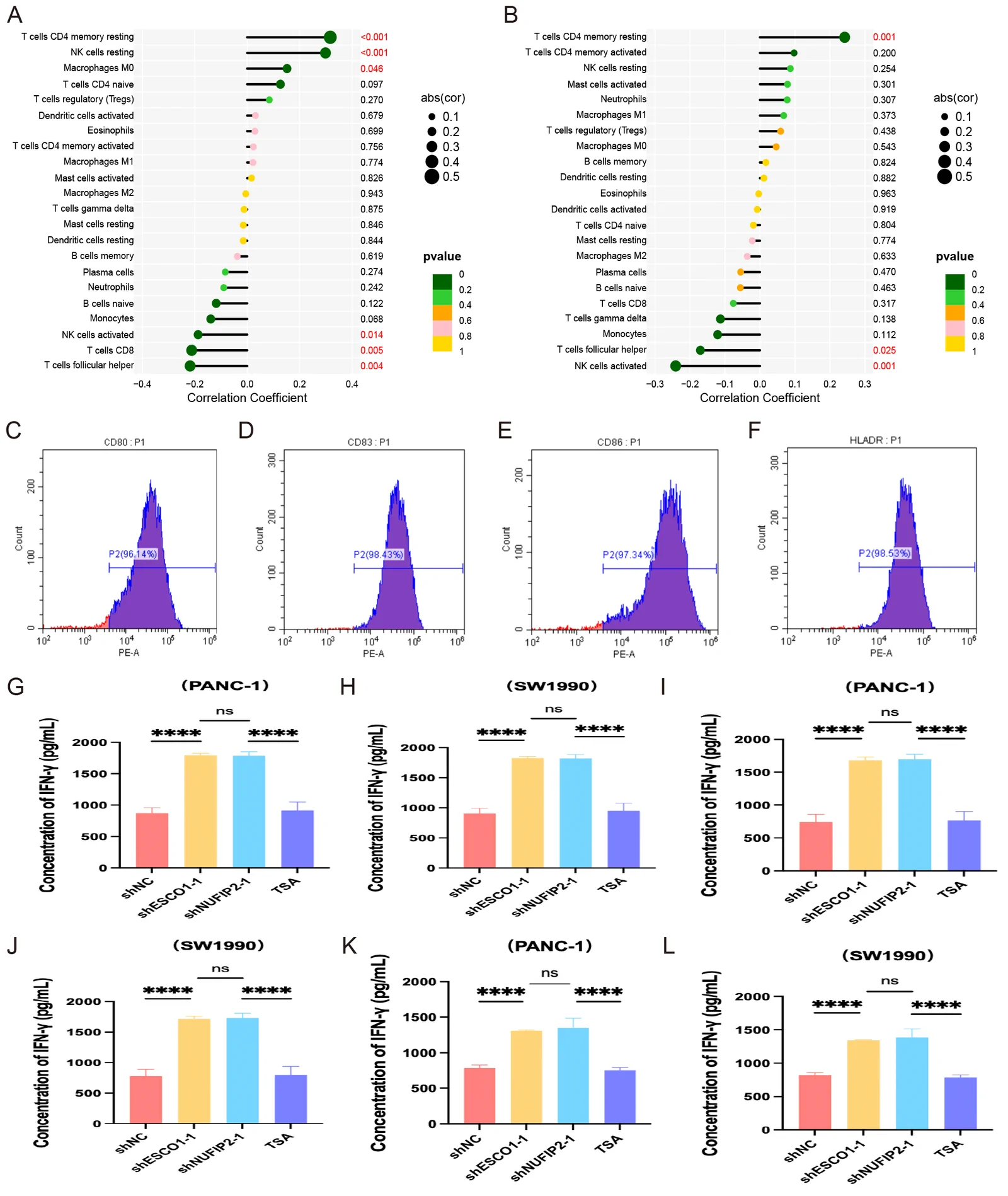
The ESCO1/NUFIP2 axis modulates antitumor immune responses in pancreatic cancer. **(A, B)** Correlations of ESCO1 and NUFIP2 expression with tumor-infiltrating immune cells. **(C–F)** Flow cytometric analysis of CD80, CD83, CD86, and HLA-DR expression in induced DCs. **(G–L)** IFN-γ secretion by CTLs stimulated with DCs pulsed with tumor lysates from PANC-1 and SW1990 cells subjected to ESCO1 or NUFIP2 knockdown, or ESCO1 knockdown combined with TSA treatment. ****P < 0.0001.

To further evaluate the impact of the ESCO1/NUFIP2 axis on antitumor immune responses, dendritic cells (DCs) and T cells were induced *in vitro*. Flow cytometric analysis showed high expression of CD80, CD83, CD86, and HLA-DR on the induced DCs (**Figures 13C–F**), confirming a mature DC phenotype. DCs were subsequently pulsed with tumor cell lysates derived from differently treated PANC-1 and SW1990 cells. The lysate-pulsed DCs were then used to stimulate T cells and generate cytotoxic T lymphocytes (CTLs), allowing us to assess the effects of manipulating the ESCO1/NUFIP2 axis in tumor cells on CTL-mediated antitumor immune responses.

ELISA showed that CTLs stimulated by DCs pulsed with lysates from ESCO1- or NUFIP2-knockdown tumor cells secreted significantly higher levels of IFN-γ than those stimulated by DCs pulsed with lysates from shNC cells (**Figures 13G–L**), indicating that depletion of ESCO1 or NUFIP2 in tumor cells enhances DC-mediated CTL responses. Notably, TSA treatment of ESCO1-knockdown tumor cells significantly reduced IFN-γ secretion by the resulting CTLs, partially reversing the increase induced by ESCO1 depletion. These findings suggest that restoration of acetylation levels can partially reverse the enhanced CTL response caused by ESCO1 loss.

## 4 Discussion

Pancreatic cancer is one of the most aggressive malignancies of the digestive system and is associated with a dismal prognosis. Its development and progression are driven by multilayered molecular alterations involving transcriptional dysregulation, post-translational modifications, disrupted protein homeostasis, mitochondrial dysfunction, and remodeling of the tumor immune microenvironment (21, 22). Despite substantial advances in our understanding of the molecular landscape of pancreatic cancer, how these regulatory alterations interact and cooperate to sustain malignant progression remains incompletely understood (23). In the present study, we identified the ESCO1/NUFIP2 axis as a previously unrecognized oncogenic regulatory pathway and uncovered a potential mechanistic link between post-translational regulation, protein homeostasis, mitochondrial apoptosis, and antitumor immunity. These findings broaden the biological relevance of ESCO1 and NUFIP2 in cancer and provide new insights into the interplay between tumor cell-intrinsic survival programs and immune regulation during pancreatic cancer progression.

ESCO1 is an acetyltransferase and chromosome cohesion regulator whose canonical functions include sister chromatid cohesion, DNA replication, and maintenance of genomic stability (24). With growing recognition of the importance of post-translational modifications in tumorigenesis and cancer progression, the potential oncogenic functions of ESCO1 have attracted increasing attention (25). Our pan-cancer analysis revealed aberrant ESCO1 expression across multiple tumor types and demonstrated associations with patient prognosis and immune infiltration. Given the aggressive nature and poor prognosis of pancreatic cancer, together with the limited understanding of ESCO1 in this malignancy, we further investigated its biological significance and underlying mechanisms in pancreatic cancer. Functional assays demonstrated that ESCO1 depletion markedly suppressed pancreatic cancer cell proliferation, colony formation, and migration while promoting apoptosis. Collectively, these findings support an oncogenic role for ESCO1 in pancreatic cancer and extend its biological significance beyond its canonical function in chromosome cohesion.

Having established the oncogenic role of ESCO1, we subsequently identified NUFIP2 as a potential downstream effector. NUFIP2 has traditionally been characterized as an RNA-binding protein involved in RNA granule transport, RNA localization, and RNA homeostasis, whereas its contribution to tumorigenesis and cancer progression remains poorly understood (26). In the present study, NUFIP2 was aberrantly upregulated in pancreatic cancer and was closely associated with disease progression and unfavorable clinical outcomes. Functionally, NUFIP2 depletion markedly impaired pancreatic cancer cell proliferation and migration while promoting apoptosis. These observations suggest that the biological role of NUFIP2 in pancreatic cancer may extend beyond its established functions in RNA homeostasis and encompass the maintenance of malignant cellular phenotypes. Notably, NUFIP2 exhibited oncogenic phenotypes consistent with those of ESCO1, and its protein abundance was strongly influenced by ESCO1, suggesting that NUFIP2 may function as an important downstream effector rather than merely an ESCO1-associated molecule.

We therefore sought to define the molecular relationship between ESCO1 and NUFIP2. Correlation analysis, molecular docking, Co-IP, and immunofluorescence collectively supported a close molecular association and physical interaction between the two proteins. More importantly, ESCO1 regulated NUFIP2 predominantly at the level of protein stability rather than mRNA expression. ESCO1 depletion accelerated NUFIP2 degradation, shortened its protein half-life, and increased its ubiquitination. Given the established role of K48-linked ubiquitination in proteasome-dependent degradation (27), together with our K48-linked ubiquitination data, these findings support a model in which ESCO1 preserves NUFIP2 protein stability by limiting its K48-linked ubiquitination and subsequent degradation. Thus, the relationship between ESCO1 and NUFIP2 extends beyond expression correlation and physical interaction to the regulation of NUFIP2 protein fate, providing a mechanistic explanation for its aberrant accumulation in pancreatic cancer.

Of particular interest, ESCO1-mediated regulation of NUFIP2 stability appears to involve crosstalk between acetylation and ubiquitination. Both modifications can occur on lysine residues and may influence one another through competition for modification sites, conformational changes, or altered recognition by E3 ubiquitin ligases (28). ESCO1 depletion reduced NUFIP2 acetylation while enhancing its ubiquitination. Conversely, treatment of ESCO1-depleted cells with the deacetylase inhibitor trichostatin A (TSA) partially restored NUFIP2 acetylation and attenuated its ubiquitination. These findings suggest that ESCO1-mediated acetylation may represent a critical molecular mechanism linking ESCO1 activity to the ubiquitination status and protein fate of NUFIP2. This observation expands the functional scope of ESCO1 as an acetyltransferase in cancer and provides further evidence for functional crosstalk between distinct post-translational modifications in pancreatic cancer. However, because the specific acetylation sites on NUFIP2 remain unidentified, whether acetylation and ubiquitination directly compete for the same lysine residues remains to be determined.

Mitochondrial dysfunction is a fundamental feature of cancer biology that contributes to apoptosis evasion, adaptation to metabolic stress, and therapeutic resistance (29). As a central regulatory platform for intrinsic apoptosis, mitochondrial disruption can lead to loss of membrane potential, increased oxidative stress, and cytochrome c (Cyt c) release, ultimately triggering caspase activation and apoptotic cell death. Pancreatic cancer cells may acquire a survival advantage under adverse conditions, including hypoxia, nutrient deprivation, and therapeutic stress, by preserving mitochondrial function and increasing the threshold for apoptosis (30). Notably, Gene Ontology (GO) enrichment analysis of NUFIP2-associated genes highlighted processes related to apoptotic mitochondrial changes, providing an initial indication that NUFIP2 may participate in the regulation of mitochondrial function and intrinsic apoptosis. Consistent with this bioinformatic evidence, depletion of either ESCO1 or NUFIP2 resulted in loss of mitochondrial membrane potential and accumulation of mitochondrial reactive oxygen species (mtROS). ESCO1 depletion also promoted Cyt c release, disrupted the BCL2/BAX balance, and activated Caspase-9 and Caspase-3, collectively indicating activation of the mitochondrial apoptotic pathway (31). Importantly, restoration of NUFIP2 expression partially reversed the apoptosis-related alterations induced by ESCO1 depletion, whereas TSA treatment partially alleviated the associated mitochondrial dysfunction. Together with the preceding evidence that ESCO1 regulates NUFIP2 acetylation and protein stability, these findings position NUFIP2 as a potential molecular link between ESCO1-mediated post-translational regulation and mitochondrial apoptosis. The ESCO1/NUFIP2 axis may therefore confer a survival advantage on pancreatic cancer cells by preserving mitochondrial function and restraining intrinsic apoptosis.

Beyond its tumor cell-intrinsic effects, the ESCO1/NUFIP2 axis may also contribute to the regulation of antitumor immunity in pancreatic cancer. Pancreatic cancer is characterized by a profoundly immunosuppressive microenvironment, in which impaired effector T-cell and NK-cell activity, together with complex tumor–stromal interactions, contributes to immune evasion (32). In our study, ESCO1 and NUFIP2 expression was associated with distinct immune infiltration patterns that were broadly indicative of reduced antitumor immune activity. Single-cell and cell–cell communication analyses further suggested that ESCO1/NUFIP2-associated tumor cells may participate in complex signaling networks involving immune and stromal populations. These findings raise the possibility that the biological effects of the ESCO1/NUFIP2 axis extend beyond tumor cell-autonomous processes to influence interactions between malignant cells and the surrounding immune microenvironment.

This possibility was further supported by our *in vitro* immune functional assays. Depletion of either ESCO1 or NUFIP2 enhanced CTL responses induced by dendritic cells (DCs) sensitized with tumor cell lysates, whereas TSA treatment partially reversed the effect of ESCO1 depletion. These findings suggest that the ESCO1/NUFIP2 status of tumor cells and its associated acetylation-dependent regulation may influence DC–CTL-mediated antitumor immune responses. Nevertheless, the precise mechanisms underlying this immunomodulatory effect remain to be elucidated.

From a translational perspective, characterization of the ESCO1/NUFIP2 axis provides a potential basis for biomarker development and therapeutic intervention in pancreatic cancer. The aberrant expression of ESCO1 and NUFIP2, together with their associations with disease progression and clinical outcomes, supports further evaluation of their potential as candidate biomarkers. More importantly, the ESCO1-mediated regulation of NUFIP2 acetylation and protein stability identifies several potentially actionable molecular events. ESCO1 acetyltransferase activity, the ESCO1–NUFIP2 interaction, and key post-translational modifications of NUFIP2 may each represent potential points of therapeutic intervention. Further elucidation of the structural basis and critical modification sites underlying this regulatory axis, together with the development of more selective approaches to disrupt its activity, may therefore open new avenues for mechanism-based therapeutic strategies in pancreatic cancer.

Several limitations of the present study should be acknowledged. First, the key acetylation and ubiquitination sites on NUFIP2 remain undefined. Consequently, direct site-specific evidence explaining how ESCO1-mediated acetylation influences K48-linked ubiquitination of NUFIP2 is still lacking. Future studies combining mass spectrometry, site-directed mutagenesis, and catalytically deficient ESCO1 mutants will be necessary to establish the molecular basis of this regulatory mechanism. Second, TSA is a broad-spectrum deacetylase inhibitor, and its rescue effects may involve additional acetylated substrates and signaling pathways. More specific genetic and biochemical approaches are therefore required to establish the causal contribution of ESCO1-mediated NUFIP2 acetylation to the observed phenotypes. Third, ESCO1 expression in our clinical cohort was primarily validated by Western blotting, and its tissue-level distribution remains to be further characterized. Future studies incorporating larger clinical cohorts and immunohistochemical analyses are warranted to validate the spatial expression of ESCO1 in pancreatic cancer. Finally, although immune infiltration analysis, single-cell profiling, and DC–CTL functional assays collectively suggest that the ESCO1/NUFIP2 axis may participate in antitumor immune regulation, its underlying mechanisms and functional relevance within an intact immune microenvironment remain unclear. Future studies incorporating spatially resolved tissue analyses and immunocompetent animal models will be important for defining the role of this axis in tumor–immune interactions and evaluating its potential therapeutic relevance.

Overall, our findings expand the biological roles of ESCO1 and NUFIP2 in pancreatic cancer and establish a potential connection between post-translational regulation, mitochondrial apoptosis, and antitumor immunity. Further investigation of the ESCO1/NUFIP2 axis may deepen our understanding of the molecular mechanisms underlying pancreatic cancer progression and provide a rationale for the development of mechanism-based therapeutic strategies.

## 5 Conclusion

This study identifies the ESCO1/NUFIP2 axis as a previously unrecognized oncogenic regulatory pathway in pancreatic cancer. Mechanistically, ESCO1 promotes NUFIP2 acetylation and limits its K48-linked ubiquitination and subsequent degradation, thereby maintaining NUFIP2 protein stability. Stabilized NUFIP2 preserves mitochondrial function, restrains intrinsic apoptosis, and promotes pancreatic cancer progression. Moreover, the ESCO1/NUFIP2 axis is associated with immune cell infiltration and may contribute to the regulation of DC-mediated CTL antitumor responses. Collectively, these findings establish a mechanistic link between post-translational regulation, protein homeostasis, mitochondrial apoptosis, and antitumor immunity, and support further investigation of the ESCO1/NUFIP2 axis as a potential biomarker and therapeutic target in pancreatic cancer.

## Data Availability

The original contributions presented in the study are included in the article/supplementary material. Further inquiries can be directed to the corresponding author.
